# Associations Between Maternal Polychlorinated Biphenyls (PCBs) Exposure from Seafood Consumption during Pregnancy and Lactation and Child Growth: A Systematic Review and Meta-Analysis

**DOI:** 10.1016/j.advnut.2024.100350

**Published:** 2024-11-30

**Authors:** Arin A Balalian, Maureen K Spill, Rachel C Thoerig, Rupal Trivedi, Sanjoy Saha, Margaret J Foster, Amanda J MacFarlane

**Affiliations:** 1 Texas A&M Agriculture, Food and Nutrition Evidence Center, AgriLife Research, TX, United States; 2 Center for Systematic Reviews and Research Syntheses, University Libraries, Texas A&M University, TX, United States; 3 Department of Nutrition, Texas A&M University, TX, United States

**Keywords:** polychlorinated biphenyls, PCB, child growth, birthweight, pregnancy, lactation

## Abstract

Beyond its nutritional benefits, seafood is a source of toxicant exposure including polychlorinated biphenyls (PCB). The association of PCB exposure from seafood intake during pregnancy and/or lactation (PL) and child growth outcomes is uncertain. This systematic review investigated the evidence and quantified the association between PCB exposure during PL from seafood intake and child growth outcomes. Embase, PubMed, and CENTRAL databases were searched from their inception for peer-reviewed English articles. Records were screened independently by 2 researchers at title and abstract, and then full-text levels. Studies were included if they: *1*) were conducted in a country with a high Human Development Index, *2*) measured maternal PCB exposure directly, *3*) assessed the relationship between PCB and seafood exposures or PCB or seafood associations with a child growth outcome, and *4*) were randomized or nonrandomized interventions, cohort, or nested case-control studies. Pooled partial correlations (*r*_*p*_) were calculated using random-effects models for studies with sufficient data and narratively for the remaining studies. Cochrane ROBINS-E and GRADE tools were used to assess risk of bias and certainty of evidence, respectively. Child growth outcomes included birthweight, birth length, head and chest circumference at birth, and small for gestational age (SGA). Seven studies were included. PCB exposure during PL was weakly but significantly associated with lower birthweight [*r*_*p*_ = −0.07; 95% confidence interval (CI): −0.12, −0.02; *n* = 5], but showed no association with birth length (*r*_*p*_ = −0.04; 95% CI: −0.09, 0.02; *n* = 4) and head circumference (*r*_*p*_ = −0.03, 95% CI: −0.09, 0.03; *n* = 3). Studies on SGA and chest circumference yielded inconclusive results. The certainty of the evidence was low or very low because of the risk of bias from confounding, missing data, and exposure misclassification. The evidence suggests minimal to no link between PCB exposure from seafood during PL on child growth outcomes but with low to very low certainty.

This trial was registered at PROSPERO as CRD42023493302.

**Statement of Significance:**

This systematic review assessed the association between exposure to PCB from seafood during pregnancy and child growth outcomes. We found minimal to no association between maternal PCB exposure from seafood and child birthweight, birth length, head and chest circumference at birth, and small for gestational age. The certainty of the evidence is low to very low because of study design and risk of bias.

## Introduction

Child growth and development are influenced by maternal exposures, both beneficial and detrimental, during pregnancy and/or lactation (PL) [1]. Seafood, defined as fish and shellfish, is a source of essential macro- and micronutrients required for optimal fetal and child growth [2]. It provides essential nutrients that play integral roles in fundamental biological processes [1,3]. However, seafood is also a potential route of exposure to various environmental contaminants, including polychlorinated biphenyls (PCB) [4]. Thus, the United States Food and Drug Administration has suggested limiting seafood consumption to 12 oz/wk during PL to balance the benefits of nutrients and risks from pollutants [5].

PCBs are synthetic chemicals with dielectric and coolant properties that were used in transformers, capacitors and large appliances [6]. Although PCBs were banned in the 1970s in the United States, they persist in the environment, including the oceans and seas [7]. They bioaccumulate in seafood that now represents a primary source of human PCB exposure. PCBs can cross the placenta, potentially impacting fetal development [[8], [9], [10], [11]]. The prenatal period is a critical window for brain development, during which cognitive, motor, and behavioral functions are established [12]. Because of the immaturity of the blood–brain barrier and incomplete development of compensatory mechanisms, the fetal brain is particularly vulnerable to toxic environmental exposures [[13], [14], [15], [16]]. Previous studies including systematic reviews have shown that perinatal exposure to PCBs is associated with adverse neurodevelopmental outcomes such as lower scores on cognitive development, motor development, and attention related outcomes [17,18]. Moreover, owing to their lipophilic nature, PCBs accumulate in adipose tissue and are excreted through human milk [[19], [20], [21], [22]], thereby exposing newborns and infants to these compounds.

A recent National Academies of Sciences, Engineering, and Medicine committee was charged with examining the role of seafood consumption on child growth and development, with consideration for both nutritional benefits and adverse effects because of contaminant exposure [23]. In service of this committee, we completed a scoping review to estimate the scope of literature related to exposure to various toxicants from seafood consumption during PL and childhood on child outcomes [24]. We identified PCB exposure during PL and child growth outcomes as 1 of only a few toxicant–outcome pairs with sufficient evidence to conduct a systematic review [24]. A systematic review examining the relationship between PCB, specifically from seafood consumed during the PL period, and child growth outcomes will provide important information for policymakers tasked with evaluating current seafood dietary recommendations.

## Methods

The systematic review included a meta-analysis with a summary of pooled statistics using partial correlation coefficients. We narratively described the evidence regarding the outcomes where there was not sufficient data to calculate partial correlation coefficients. We followed the “Preferred Reporting Items for Systematic Review and Meta-Analyses” (PRISMA), and the Meta-analysis of Observational Studies in Epidemiology (Supplemental Table 1a and b) guidelines and registered the protocol in PROSPERO (CRD42023493302).

A search strategy was developed by an academic librarian (MF) based on the Population, Exposure, Comparison group, Outcome and Study design (PECOS) framework and eligibility criteria. Embase, PubMed, and CENTRAL databases were searched for peer-reviewed articles published in English before December 21, 2023. The detailed search strategy is described in Supplemental Table 2.

We used the PECOS framework to determine the eligibility of articles to be included in this systematic review (Supplemental Figure 1). The inclusion and exclusion criteria were developed in consultation with experts from the National Academies of Sciences, Engineering, and Medicine Committee [23] and are summarized in Supplemental Table 3. Briefly, studies had to take place in countries classified as high or very high on the Human Development Index (HDI), as this is the criteria for systematic reviews that inform the Dietary Guidelines for Americans [25]. Studies were required to assess both seafood consumption and PCB exposure during the PL period. Additionally, studies had to evaluate the association between PCB and seafood, as well as each exposure independently with growth outcomes. Alternatively, studies were eligible if they assessed the associations of both PCB and seafood with growth outcomes if the relationship between seafood and PCB was not directly reported (Supplemental Figure 2). Moreover, studies needed to compare PCB exposure across different concentrations, including no exposure. For seafood intake, studies had to compare consumption based on different types, sources, amounts, frequencies, durations, methods of preparation, or timings of consumption, including no seafood intake. Eligible study designs included prospective and retrospective cohort studies, case-cohort studies, case-control studies, before-and-after studies, quasi-experimental designs, and randomized controlled trials. Records were screened by 2 independent screeners at the title and abstract, followed by full-text levels using DistillerSR software [26]. Disagreements were reviewed and resolved, if necessary, by consulting a third reviewer. Additionally, the reference lists in the included full-text studies were searched manually to include any potentially relevant articles not identified in the database search.

All data were extracted by an experienced, trained analyst using a standard extraction form and checked for accuracy and completeness by a second analyst. Extracted data fields included information about study characteristics, study population, toxicant exposure and seafood intake, child growth outcomes, statistical analysis, and summary of results.

All studies underwent dual, independent risk of bias (ROB) assessments using a tool designed specifically for nonrandomized exposure studies (ROBINS-E) [27]. All 7 domains within the tool were assessed and considered for an overall ROB rating for each article. Conflicts were reviewed and resolved by a third reviewer if necessary. Certainty of evidence was determined using the GRADE approach [28].

For quantitative synthesis, pooled partial correlations (*r*_*p*_) were calculated following the methods of Aloe and Thompson [29] (Supplemental Method 1). We used the *rma* function in the metafor [30] and metadat [31] packages using R 4.3.2 [32] to fit random-effects models to pool the effect estimates. We determined the strength of correlation coefficients on the basis of previously suggested guidance [33]. For all other outcomes, we conducted a qualitative synthesis.

We explored effect-size heterogeneity and risk of publication bias using *Q* and *I*^2^. *I*^2^ of 0%–40% were interpreted as low, 30%–60% as moderate, 50%–90% as substantial, and 75%–100% as considerable heterogeneity, according to the Cochrane’s guidebook [34]. Funnel plots were inspected to detect publication bias.

The effect estimates with highest magnitude were used to calculate the partial correlations if >1 effect estimate was available per study [35]. We used the effect estimates for dioxin-like PCBs (DL-PCBs) for 1 study [36] where multiple analyses were conducted on the basis of PCB structure or their hypothesized mechanism of action [37]. In a sensitivity analysis, we investigated whether choice of PCB influenced the results (Supplemental Method 2). Finally, in an additional analysis, we removed the studies [35,38] in which the association between PCB exposure and seafood consumption was not directly assessed.

## Results

### Overall study characteristics

**FIGURE 1:**
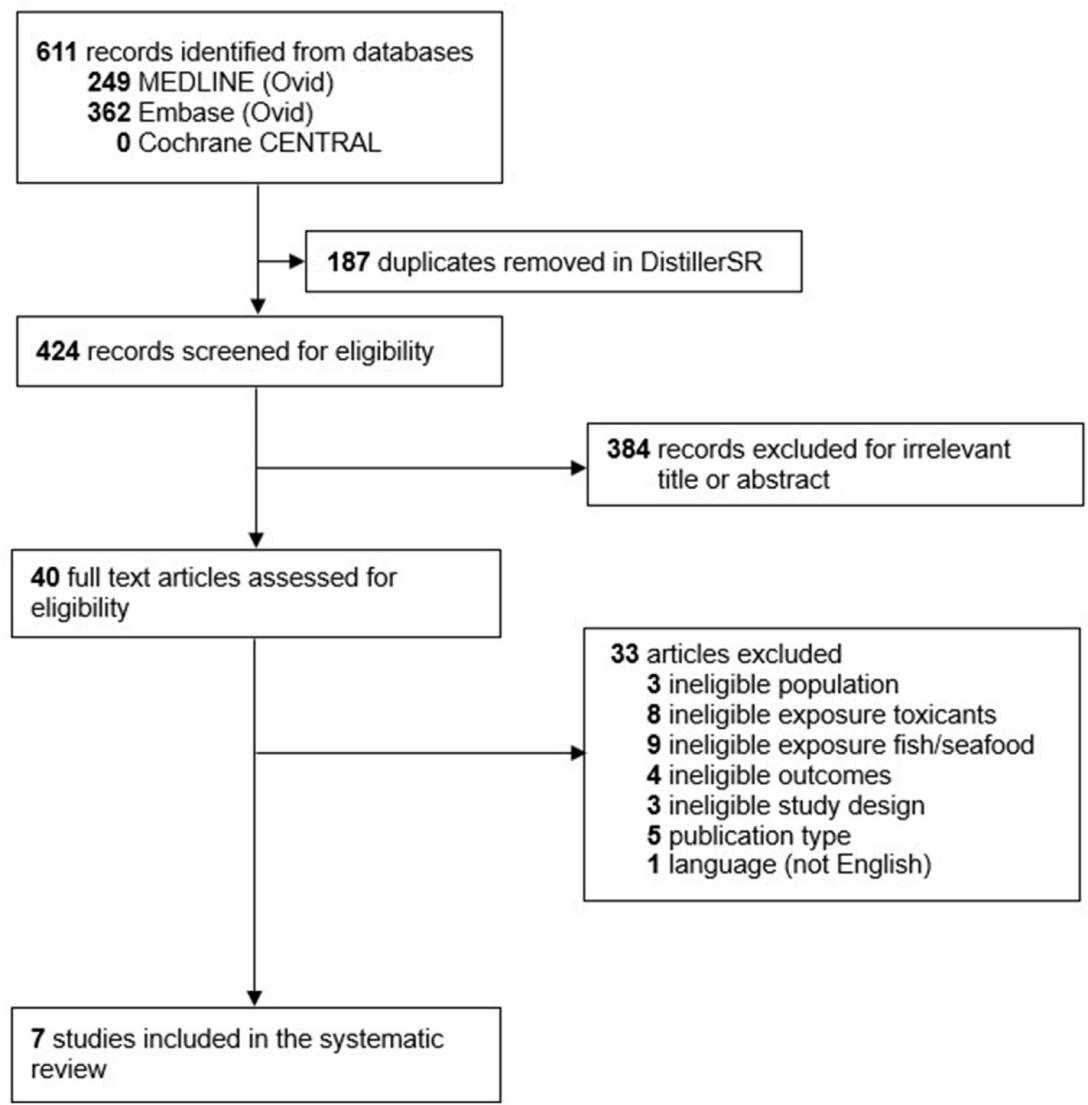
Preferred Reporting Items for Systematic Review and Meta-Analyses (PRISMA) flow diagram of screened and selected studies.

Overall, 611 records were identified with 7 articles included in the systematic review after duplicate removal and screening (Figure 1). All the articles originated from 7 different prospective cohort studies from the United States, Faroe Islands, and Denmark (Table 1) [26,35,36,[38], [39], [40], [41], [42]], where exposure assessment was conducted before outcome evaluation, with no cross-sectional analyses.

**TABLE 1:** Characteristics and results of included studies.

Study identifier; name of the study/cohort; country	PCB exposure assessment mode and time; PCB congeners tested	PCB concentrations (unit)	Results (PCB and growth outcomes)	Findings summary (PCB and growth outcomes)
Birthweight and postnatal weight gain				
Fein (1984) [41]; Lake Michigan Cohort Study; United States	Cord blood at birth Congeners: NR	Not reported	Birth weight (kg) mean (SD), (*n* = 313) PCB levels <LOD (3 ng/mL): 3.57 (0.54) PCB levels ≥LOD (3 ng/mL): 3.41 (0.54) *P* value < 0.05	Infants exposed to higher PCB concentrations had on average significantly higher birthweight compared with infants exposed to lower PCB concentrations
Grandjean (2001); [38] Faroe Island cohort study; Faroe Islands	Maternal serum at 34th wk gestation; Congeners: Sum of 28 congeners^1^	∑PCB GM (IQR) 0.86 (1.05) μg/g lipid	β (SE), (*n* = 182) −31.0 (99.9) *P* value = 0.76 Birthweight (mean) (g) PCB <0.6 ng/g lipid: 3691 PCB 0.6–1.3 ng/g lipid: 3557 PCB >1.3 ng/g lipid: 3606 *P* value = 0.47	Results indicate a nonsignificant negative association between perinatal exposure to higher concentrations of PCBs and lower birthweight.
Halldorsson (2008); [40] Danish National Birth Cohort; Denmark	Maternal blood plasma at eighth and 25th wk gestation; Congeners: 105, 118, 138, 153, 156, 180	∑PCB Median (IQR): 1.15 μg/L (0.91, 1.45)	Birth weight (g): β (95% CI), (*n* = 100) –334 (–628, –40), *P* value = 0.03	Maternal plasma PCBs concentration had a significant and negative association with birth weight.
Mendez (2010); [39] 1 INfancia y Medio Ambiente: ‘INMA’; Spain	Maternal serum from the end of first trimester-beginning of second trimester; Congeners: NR;	GM: PCB 138: 0.10 ng/ml PCB 153: 0.18 ng/ml PCB 180: 0.12 ng/ml	Birthweight (g) β (95% CI), (*n* = 592) All seafood ≤=3 servings/wk Ref. >3–6 servings/wk: –20.8 (–119.0, 77.4) >6 servings/wk: –64.5 (–162.2, 33.1) Crustaceans >1/wk: –59.1 (–143.2, 24.9) Other shellfish >1/wk: –51.9 (–121.3, 17.5) Fatty fish >1/wk: 0.1 (–61.8, 62.1) Lean fish >3/wk: –6.1 (–68.0, 55.9) Canned tuna >1/wk: –13.7 (–78.3, 51.0)	No association of seafood intake with birthweight
Miyashita (2015); [36] Hokaido Study on Environment and Children's health; Japan	Maternal blood at third trimester or within 5 d postpartum; Congeners: ∑PCB _estrogenic_ (52, 49 47, 44, 70, 95, 101, 99, 110, 153) ∑PCB _antiestrogenic_ (37, 77, 81, 126, 169, 114, 105, 156) ∑PCB _dioxin-like_ (77, 81, 105, 114, 118, 123, 126, 156, 157, 167, 169, 189) ∑PCB _non-dioxin-like_^2^	Median (IQR): ∑PCB _estrogenic_ 28.7 (19.5, 40.0) ng/g lipid ∑PCB _antiestrogenic_ 4.13 (2.75, 5.60) ng/g lipid ∑PCB _dioxin-like_ 11.2 (7.51, 15.6) ng/g lipid ∑PCB _non-dioxin-like_ 95.7 (64.8, 133) ng/g lipid	Birthweight (g) β (95% CI), (*n* = 367) Estrogenic PCBs: –80.1 (−259, 98.8) Antiestrogenic PCBs: −87.3 (−258, 83.5) Dioxin−like PCBs: −119 (−290, 52.1) Non-dioxin-like PCBs: −104 (−289, 81.4)	Results indicate a nonsignificant negative association between perinatal exposure to higher concentrations of PCBs and lower birthweight.
Sagiv (2007); [35] New Bedford Cohort Study; United States	Cord blood at birth; Congeners: ∑ of 4 PCB: (118, 138, 153, 180) ∑ of mono-ortho PCBs: (105, 118 156, 167, 189)	(min, max) ∑ of 51 PCBs: (0.07, 18.14) ng/g serum ∑ of 4 PCBs: (0.00, 4.41) ng/g serum ∑ of mono-ortho PCBs: (0, 151.49) pg/g serum PCB 118: (0, 2.05) ng/g serum	Birthweight (g) β (95% CI), (*n* = 718) ∑ of 51 PCBs: Quartile 1: Ref. Quartile 2: −16.0 (−104.5, 72.5) Quartile 3: −101.2 (−194.5, −7.9) Quartile 4: −47.6 (−152.9, 57.7) *P* for trend = 0.43 ∑ of 4 PCBs: Quartile 1: Ref. Quartile 2: −65.0 (−154.5, 24.5) Quartile 3: −95.8 (−189.6, −2.0) Quartile 4: −99.2 (−204.2, 5.8) *P* for trend = 0.13 ∑ of mono-ortho PCBs: Quartile 1: Ref. Quartile 2: 0.4 (−88.9, 89.6) Quartile 3: −44.8 (−138.9, 49.3) Quartile 4: −74.8 (−178.2, 28.5) *P* for trend = 0.10 PCB 118: Quartile 1: Ref. Quartile 2: −18.0 (−105.8, 69.9) Quartile 3: −72.0 (−164.6, 20.6) Quartile 4: −69.5 (−167.9, 29.0) *P* for trend = 0.20	Results indicate a nonsignificant negative association between perinatal exposure to higher concentrations of PCBs and lower birthweight.
Wohlfahrt-Veje, 2014; [42] Copenhagen Mother Child Cohort of Growth and Reproduction; Denmark	Human milk (1–3 mo after delivery); Congeners: Total Toxic equivalent (PCDD/PCDF + dl/PCB)	Median (min, max): TEQ 20.2. (4.9, 114.1)	Weight β (95% CI), *P* value, (*n* = 417) SDS 0 mo: −0.31 (−0.66, 0.05) *P* value ≤ 0.1 SDS 3 mo: −0.03 (−0.42, 0.37), *P* value > 0.1 SDS 18 mo: 0.26 (−0.20, 0.72) *P* value > 0.1 SDS 36 mo: 0.12 (−0.37,0.62) *P* value > 0.1 Weight change β (95% CI), *P* value, (*n* = 417) Change in weight SDS 0–3 mo: 0.22 (−0.13, 0.58), *P* value > 0.1 Change in weight SDS 0–18 mo: 0.52 (0.03,1.00), *P* value < 0.05 Change in weight SDS 0–36 mo: 0.39 (−0.12,0.91), *P* value > 0.1	Results indicate a negative association between perinatal PCB exposure and weight at birth and at 3 mo. and positive associations at 18 and 36 mo. Results indicate a positive association between perinatal PCB exposure and weight change from 0–3 mo; 0–18 mo and 0–36 mo.
Birth length, postnatal length, and height				
Halldorsson (2008); [40] Danish National Birth Cohort; Denmark	Maternal blood plasma at eighth and 25th wk gestation; Congeners: 105, 118, 138, 153, 156, 180	∑PCB Median (IQR): 1.15 μg/L (0.91, 1.45)	Birth length (cm) β (95% CI), (*n* = 100) −1.2 (−2.5, 0.2), *P* value = 0.08	Maternal plasma PCBs concentration had a nonsignificant and negative association with birth length.
Miyashita (2015); [36] Hokaido Study on Environment and Children’s Health; Japan	Maternal blood at third trimester or within 5 d postpartum; Congeners: ∑PCB _estrogenic_ (52, 49 47, 44, 70, 95, 101, 99, 110, 153) ∑PCB _antiestrogenic_ (37, 77, 81, 126, 169, 114, 105, 156) ∑PCB _dioxin-like_ (77, 81, 105, 114, 118, 123, 126, 156, 157, 167, 169, 189) ∑PCB _non-dioxin-like_^2^	Median (IQR): ∑PCB _estrogenic_ 28.7 (19.5, 40.0) ng/g lipid ∑PCB _antiestrogenic_ 4.13 (2.75, 5.60) ng/g lipid ∑PCB _dioxin-like_ 11.2 (7.51, 15.6) ng/g lipid ∑PCB _non-dioxin-like_ 95.7 (64.8, 133) ng/g lipid	OR (95% CI), (*n* = 367) Length (cm) Estrogenic PCBs: 0.28 (−0.72, 1.28) Antiestrogenic PCBs: −0.07 (−1.02, 0.89) Dioxin-like PCBs: 0.09 (−0.87, 1.05) Non-dioxin-like PCBs: 0.17 (−0.86, 1.21)	Results indicate nonsignificant associations between perinatal exposure to PCBs and lower or higher birth length.
Sagiv (2007); [35] New Bedford Cohort Study; United States	Cord blood at birth; Congeners: ∑ of 4 PCB: (118, 138, 153, 180) ∑ of mono-ortho PCBs: (105, 118 156, 167, 189)	(min, max) ∑ of 51 PCBs: (0.07, 18.14) ng/g serum ∑ of 4 PCBs: (0.00,4.41) ng/g serum ∑ of mono-ortho PCBs: (0,151.49) pg/g serum PCB: 118: (0, 2.05) ng/g serum	Birth length (cm) β (95% CI), (*n* = 718) ∑ of 51 PCBs: Quartile 1: Ref. Quartile 2: 0.15 (−0.38, 0.68) Quartile 3: −0.37 (−0.93, 0.20) Quartile 4: −0.12 (−0.76, 0.51) *P* for trend = 0.57 ∑ of 4 PCBs: Quartile 1: Ref. Quartile 2: −0.37 (−0.91, 0.16) Quartile 3: −0.44 (−1.00, 0.13) Quartile 4: −0.35 (−0.98, 0.28) *P* for trend = 0.51 ∑ of mono-ortho PCBs: Quartile 1: Ref. Quartile 2: −0.12 (−0.66, 0.41) Quartile 3: −0.29 (−0.86, 0.27) Quartile 4: −0.24 (−0.86,0.39) *P* for trend = 0.54 PCB 118: Quartile 1: 0 Quartile 2: 0.01 (−0.52,0.54) Quartile 3: −0.31 (−0.86, 0.25) Quartile 4: −0.11 (−0.71, 0.48) *P* for trend = 0.76	Results indicate a nonsignificant negative association between perinatal exposure to higher concentrations of PCBs and lower birth length.
Wohlfahrt-Veje (2014); [42] Copenhagen Mother Child Cohort of Growth and Reproduction; Denmark	Human milk after delivery; Congeners: Total Toxic equivalent (PCDD/PCDF + dl/PCB)	Median (min, max): TEQ 20.2. (4.9, 114.1)	Length (cm) β (95% CI), (*n* = 417) SDS 0 mo: –0.24 (–0.57, 0.09), *P* value >0.1 SDS 3 mo: 0.16 (–0.20, 0.52), *P* value >0.1 SDS 18 mo: 0.52 (0.10, 0.94), *P* value < 0.05 SDS 36 mo: 0.46 (–0.01,0.93), *P* value <0.1 Length change (cm) β (95% CI) (*n* = 417) Change in Length SDS 0–3 mo: 0.37 (0.11, 0.64), *P* value <0.05 Change in Height SDS 0–18 mo: 0.77 (0.34, 1.19), *P* value <0.05 Change in Height SDS 0–36 mo: 0.55 (0.08, 1.03), *P* value <0.05	Results indicate a negative nonsignificant association between perinatal PCB exposure and length at birth. Results indicate a positive association between perinatal PCB exposure and length at 3, 18, and 36 mo. Results indicate a positive association between perinatal PCB exposure and length change from 0–3 mo, 0–18 mo, and 0–36 mo.
Head circumference				
Fein (1984); [41] Lake Michigan Cohort Study; United States	Cord blood at birth; Congeners: NR	NR	Head circumference (cm) mean (SD), *n* = 241 PCB levels < LOD (3 ng/mL): 35.28 (1.18) cm PCB levels ≥ LOD (3 ng/mL): 34.63 (1.19) cm *P* value <0.001	Results indicate a significant negative association between perinatal exposure to PCBs and head circumference at birth.
Halldorsson (2008); [40] Danish National Birth Cohort; Denmark	Maternal blood plasma at eighth and 25th wk gestation; Congeners: (105, 118, 138, 153, 156, 180)	∑PCB Median (IQR): 1.15 μg/L (0.91, 1.45)	Head circumference (cm) β (95% CI), (*n* = 100) –0.8 (–1.9, 0.4), *P* value = 0.20	Results indicate a nonsignificant negative association between perinatal exposure to PCBs and smaller head circumference.
Miyashita (2015); [36] Hokaido Study on Environment and Children’s Health; Japan	Maternal blood at third trimester or within 5 d postpartum; Congeners: ∑PCB _estrogenic_ (52, 49 47, 44, 70, 95, 101, 99, 110, 153) ∑PCB _antiestrogenic_ (37, 77, 81, 126, 169, 114, 105, 156) ∑PCB _dioxin-like_ (77, 81, 105, 114, 118, 123, 126, 156, 157, 167, 169, 189) ∑PCB _non-dioxin-like_^2^	Median (IQR): ∑PCB _estrogenic_ 28.7 (19.5, 40.0) ng/g lipid ∑PCB _antiestrogenic_ 4.13 (2.75, 5.60) ng/g lipid ∑PCB _dioxin-like_ 11.2 (7.51, 15.6) ng/g lipid ∑PCB _non-dioxin-like_ 95.7 (64.8, 133) ng/g lipid	Head circumference (cm) β (95% CI), (*n* = 367) Estrogenic PCBs: −0.27 (−0.98, 0.43) Antiestrogenic PCBs: −0.23 (−0.90, 0.45) Dioxin-like PCBs: −0.16 (−0.84, 0.51) Non-dioxin-like PCBs: –0.36 (−1.09, 0.38)	Results indicate a nonsignificant negative association between perinatal exposure to PCBs and head circumference.
Sagiv (2007); [35] New Bedford Cohort Study; United States	Cord blood at birth; Congeners: ∑ of 4 PCB: (118, 138, 153, 180) ∑ of mono-ortho PCBs: (105, 118 156, 167, 189)	(min, max) ∑ of 51 PCBs: (0.07, 18.14) ng/g serum ∑ of 4 PCBs: (0.00, 4.41) ng/g serum ∑ of mono-ortho PCBs: (0, 151.49) pg/g serum PCB 118: (0, 2.05) ng/g serum	Head circumference (cm) β (95% CI), *n* = 718 ∑ of 51 PCBs: Quartile 1: Ref. Quartile 2: 0.02 (−0.28, 032) Quartile 3: −0.08 (−0.39, 0.24) Quartile 4: −0.05 (−0.41, 0.30) *P* for trend = 0.73 ∑ of 4 PCBs: Quartile 1: Ref. Quartile 2: − 0.23 (−0.53, 0.07) Quartile 3: −0.23 (−0.55, 0.08) Quartile 4: −0.25 (−0.60, 0.10) *P* for trend = 0.32 ∑ of mono-ortho PCBs: Quartile 1: Ref. Quartile 2: 0.02 (−0.28, 0.32) Quartile 3: 0.02 (−0.30, 0.33) Quartile 4: −0.23 (−0.58, 0.11) *P* for trend = 0.10 PCB 118: Quartile 1: Ref. Quartile 2: −0.11 (−0.41, 0.18) Quartile 3: −0.25 (−0.56, 0.06) Quartile 4: −0.20 (−0.53, 0.13) *P* for trend = 0.37	Results indicate a nonsignificant negative association between perinatal exposure to PCBs and smaller head circumference.
Small for gestational age				
Mendez (2010); [39] 1 INfancia y Medio Ambiente: ‘INMA’; Spain	Maternal serum from the end of first trimester-beginning of second trimester; Congeners: (138, 153, 180)	GM: PCB 138: 0.10 ng/ml PCB 153: 0.18 ng/ml PCB 180: 0.12 ng/ml	OR (95% CI), *n* = 565 All seafood < = 3 servings/wk Ref. >3–6 servings/wk: 1.91 (0.38–9.58) >6 servings/wk: 3.89 (0.82–18.59) Crustaceans >1/wk: 3.24 (1.34–7.83) Other shellfish >1/wk: 1.27 (0.56–2.89) Fatty fish >1/wk: 1.52 (0.70–3.30) Lean fish >3/wk: 0.76 (0.35–1.65) Canned tuna >1/wk: 2.39 (0.96–5.96)	Associations of >1 servings/wk consumption of Crustaceans fish and SGA and Adjusted SGA.
Miyashita (2015); [36] Hokaido Study on Environment and Children’s Health; Japan	Maternal blood at third trimester or within 5 d postpartum; Congeners: ∑PCB _estrogenic_ (52, 49 47, 44, 70, 95, 101, 99, 110, 153) ∑PCB _antiestrogenic_ (37, 77, 81, 126, 169, 114, 105, 156) ∑PCB _dioxin-like_ (77, 81, 105, 114, 118, 123, 126, 156, 157, 167, 169, 189) ∑PCB _non-dioxin-like_^2^	Median (IQR): ∑PCB _estrogenic_ 28.7 (19.5, 40.0) ng/g lipid ∑PCB _antiestrogenic_ 4.13 (2.75, 5.60) ng/g lipid ∑PCB _dioxin-like_ 11.2 (7.51, 15.6) ng/g lipid ∑PCB _non-dioxin-like_ 95.7 (64.8, 133) ng/g lipid	SGA by weight OR (95% CI), (*n* = 367) Estrogenic PCBs Quartile 1: Ref. Quartile 2: 0.56 (0.12, 2.58) Quartile 3: 0.42 (0.07, 2.41) Quartile 4: 1.88 (0.45, 7.83) *P* for trend = 0.696 Antiestrogenic PCBs Quartile 1: Ref. Quartile 2: 1.31 (0.32, 5.34) Quartile 3: 1.07 (0.21, 5.47) Quartile 4: 1.89 (0.43, 8.29) *P* for trend = 0.511 Dioxin-like PCBs Quartile 1: Ref. Quartile 2: 1.93 (0.48, 7.77) Quartile 3: 0.64 (0.10, 4.01) Quartile 4: 2.01 (0.44, 9.19) *P* for trend = 0.560 Non-dioxin-like PCBs Quartile 1: Ref. Quartile 2: 0.57 (0.12, 2.65) Quartile 3: 1.47 (0.34, 6.42) Quartile 4: 1.18 (0.23, 5.96) *P* for trend = 0.752	Results indicate higher odds of small for gestational age by weight among the children exposed to higher levels of perinatal PCBs compared with children perinatally exposed to lower levels of PCBs
			SGA by length OR (95% CI), (*n* = 367) Estrogenic PCBs Quartile 1: Ref. Quartile 2: 1.57 (0.62, 4.00) Quartile 3: 0.37 (0.11, 1.22) Quartile 4: 0.68 (0.23, 2.03) *P* for trend = 0.197 Antiestrogenic PCBs Quartile 1: Ref. Quartile 2: 1.53 (0.61, 3.84) Quartile 3: 0.50 (0.16, 1.57) Quartile 4: 0.94 (0.32, 2.73) *P* for trend = 0.550 Dioxin-like PCBs Quartile 1: Ref. Quartile 2: 1.79 (0.70, 4.56) Quartile 3: 0.62 (0.20, 1.94) Quartile 4: 0.83 (0.28, 2.48) *P* for trend = 0.260 Non-dioxin-like PCBs Quartile 1: Ref. Quartile 2: 2.02 (0.79, 5.17) Quartile 3: 0.49 (0.14, 1.66) Quartile 4: 0.88 (0.28, 2.76) *P* for trend = 0.345	Results indicate a lower odds of small for gestational age by length among the children exposed to higher levels of perinatal PCBs.
Chest circumference				
Miyashita (2015); [36] Hokaido Study on Environment and Children’s Health; Japan	Maternal blood at third trimester or within 5 d postpartum; Congeners: ∑PCB _estrogenic_ (52, 49 47, 44, 70, 95, 101, 99, 110, 153) ∑PCB _antiestrogenic_ (37, 77, 81, 126, 169, 114, 105, 156) ∑PCB _dioxin-like_ (77, 81, 105, 114, 118, 123, 126, 156, 157, 167, 169, 189) ∑PCB _non-dioxin-like_^2^	Median (IQR): ∑PCB _estrogenic_ 28.7 (19.5, 40.0) ng/g lipid ∑PCB _antiestrogenic_ 4.13 (2.75, 5.60) ng/g lipid ∑PCB _dioxin-like_ 11.2 (7.51, 15.6) ng/g lipid ∑PCB _non-dioxin-like_ 95.7 (64.8, 133) ng/g lipid	Chest circumference (cm) β (95% CI); (*n* = 367) Estrogenic PCBs: 0.38 (−0.40, 1.17) Antiestrogenic PCBs: 0.04 (−0.72, 0.79) Dioxin-like PCBs: 0.14 (−0.62, 0.89) Non-dioxin-like PCBs: 0.22 (−0.60, 1.04)	Results indicate a nonsignificant positive association between perinatal exposure to PCBs and chest circumference.

Abbreviations: PCB, polychlorinated biphenyls; GM, geometric mean; LOD, limit of detection; NR, not reported; OR, odds ratio; PCDDs, polychlorinated dibenzo-p-dioxins PCDFs, polychlorinated dibenzofurans; PCBs, polychlorinated biphenyls; SD, standard deviation.

1 Higher seafood intake was associated with higher PCB levels in Mendez (2010) [26] (eTable 4).

2 Name of the congeners not reported.

Exposure to PCBs was assessed during pregnancy (first to third trimester) in 3 studies [[38], [39], [40]]. One study assessed PCB concentrations in maternal blood in the third trimester or within 5 d postpartum [36]. Two studies assessed PCB concentrations at birth [35,41]. Only 1 study investigated the associations between PCBs measured in human milk, and collected 1–3 mo postpartum and child growth outcomes longitudinally [42]. PCB concentrations were assessed in cord blood in 2 studies [35,41], and in maternal blood [40] or serum [38,39] during pregnancy in 3 studies (Table 1).

The studies reported different estimates (geometric mean, median, and arithmetic mean) and units (lipid standardized compared with absolute values) to report the PCB concentrations (Table 1). In 3 studies [,39,41], seafood intake was assessed using questionnaires during pregnancy. In the remaining 4 studies, seafood intake during pregnancy was collected using questionnaires at birth[41], within 5 d postpartum [36] and within 2 wk postpartum [35,38]. No studies measured seafood intake during lactation. Only 2 studies used validated questionnaires to assess seafood intake[39,40]. The association between seafood intake and PCB concentrations was assessed in 5 studies [36,[39], [40], [41], [42]]. In all 5 studies, seafood intake during pregnancy was significantly associated with higher PCB concentrations measured at birth[41], during pregnancy [39,40], within a few days postpartum [36], or during lactation [42] (Supplemental Table 4). Two studies [35,38] did not report the association between seafood intake during PL with PCB concentrations.

Birthweight (BW) was measured in all 7 included studies [35,36,[38], [39], [40], [41], [42]]. Birth length (BL) [35,36,40,42] and head circumference (HC) [35,36,40,41], measured at birth were each documented in 4 studies. Other outcomes included small for gestational age (SGA) by weight and by length (2 studies) [36,39] and chest circumference (CC) (1 study) [36]. One study assessed the change in weight and change in length/height longitudinally from birth to 36 mo [42].

### Birthweight

**FIGURE 2:**
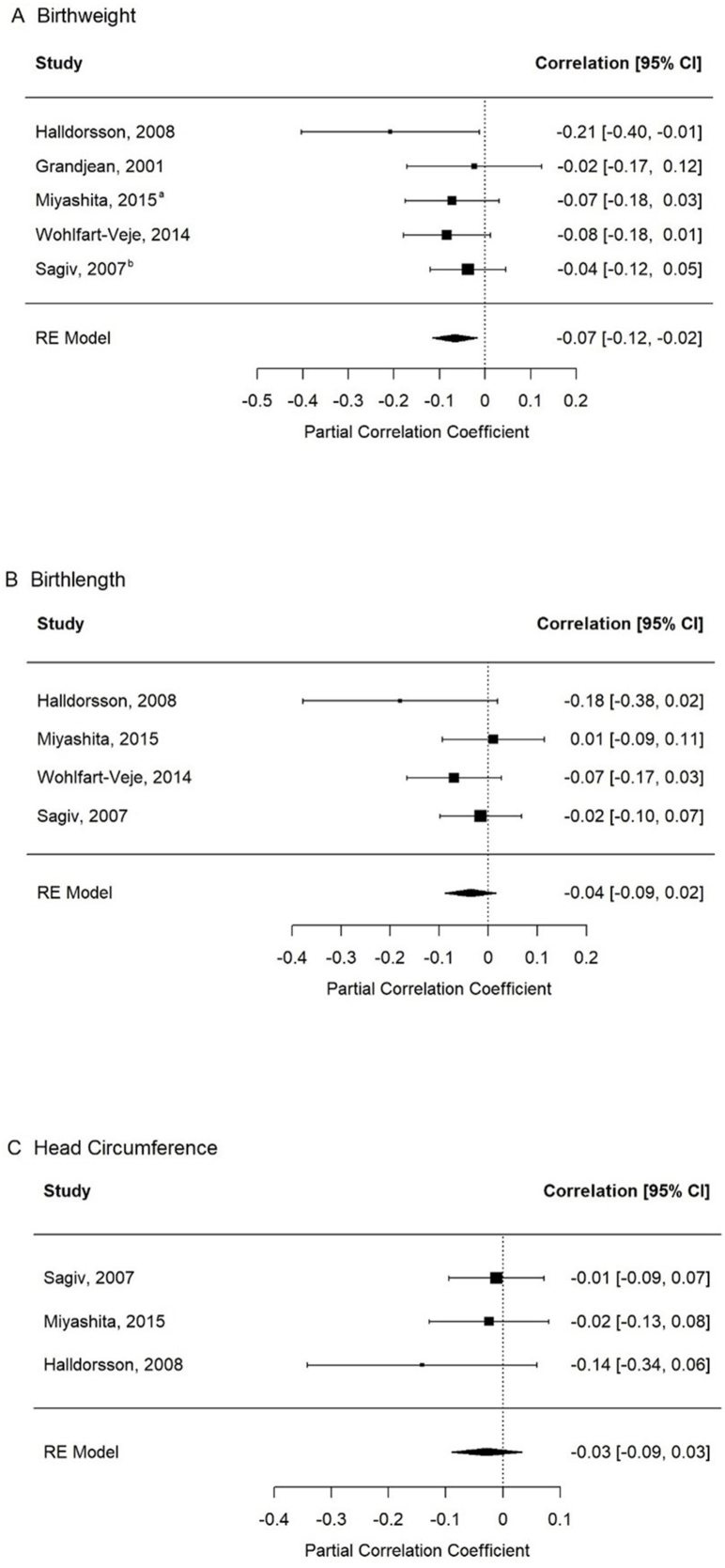
Pooled analysis of partial correlation coefficients from studies investigating the association between perinatal PCB and growth outcomes. CI, confidence interval; PCB, polychlorinated biphenyls; RE, random-effects. ^a^The effect estimates from the regression analysis for the fourth quartile compared with first quartile of PCB concentration and BW was used to calculate the partial correlation coefficient. ^b^The effect estimates from the regression analysis for dioxin-like PCBs and birthweight were used to calculate the partial correlation coefficient.

The regression data for PCB and BW association was available from 5 studies [35,36,38,40,42], and thus were synthesized quantitatively. The *r*_*p*_ analysis revealed a negative correlation between PCB exposure during pregnancy and BW partialing out the correlations between other covariates and BW [*r*_*p*_ = −0.07; 95% confidence interval (CI): −0.12, −0.02] (Figure 2A). The findings regarding exposure to other PCB groups during pregnancy and BW were similar in sensitivity analyses (data not shown). The findings were not different when we removed the studies [35,38] in which the association between PCB exposure and seafood consumption was not directly assessed. The *I*^2^ test revealed nonsignificant low heterogeneity among the studies (*Q*_df4_ = 2.94, *P* = 0.57; *I*^2^ = 7%). We did not detect any evidence of publication bias (Supplemental Figure 3).

Two studies were not included in calculating partial correlations because of the absence of necessary data to calculate *r*_*p*_ [39,41]. One study reported a lower mean BW among infants exposed to higher compared with lower cord blood concentrations of PCB (Table 1) [41]. The final study [39] did not report a direct analysis between PCB and BW but found that higher fish intake was significantly associated with higher maternal serum PCB concentrations. Higher maternal fish intakes were also associated with lower BW though the association was not statistically significant.

Overall, the evidence suggests an association between PCB exposure from fish intake during pregnancy and lower BW, but the magnitude of the effect is small. Six studies were at high ROB whereas 1 was at very high risk (Table 2) [35,36,[38], [39], [40], [41], [42]]. The certainty of the evidence was low given the very serious ROB because of inadequate control for confounding, absence of adequate measures to account for selection bias, missing data, and selective reporting of findings in included observational studies (Table 3) [35,36,[38], [39], [40], [41], [42]].

**TABLE 2:**
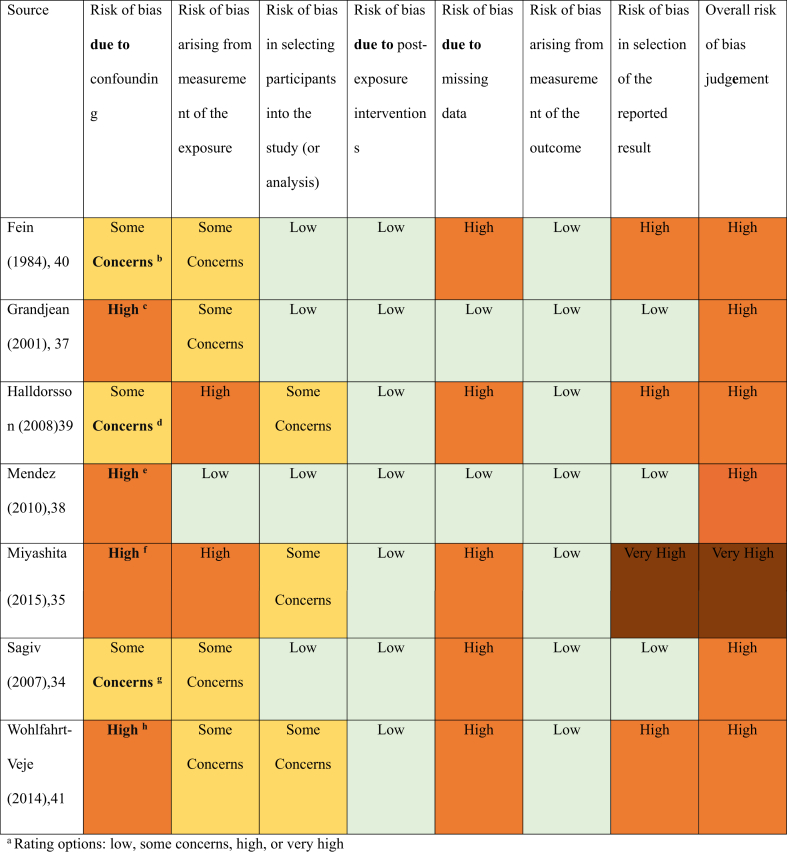

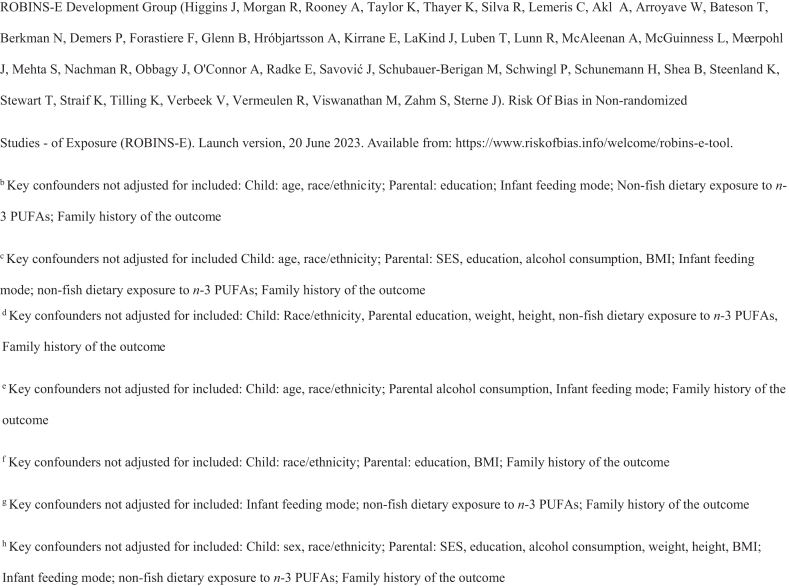
Risk of bias assessment^1^..

**TABLE 3:** GRADE^1^ assessment for evidence about relationships between perinatal exposure to polychlorinated biphenyls from seafood and child growth outcomes.

No. of articles; Author (y)	Risk of bias^2^	Inconsistency^3^,^4^	Indirectness^3^	Imprecision^3^,^5^	Publication bias^6^,^7^	Summary of findings^8^	Certainty^9^
Birthweight							
Meta-analysis: 5; Grandjean (2001), [38] Halldorsson (2008) [40] Miyashita (2015) [36] Sagiv (2007) [35] Wohlfahrt-Veje (2014) [42] Narrative synthesis: 2^10^; Fein (1984); [41] Mendez (2010) [39]	Very serious; All studies were rated as having high or very high risk of bias particularly because of confounding; selection bias from missing data; exposure measurement bias and selective reporting of data and findings	Not serious	Not serious	Not serious	Undetected	The evidence from pooled analysis suggests a negative association between Maternal PCB measured during pregnancy/lactation and birthweight. *r*_*p*_ = –0.07 (95% CI: –0.12, –0.02) The evidence from narrative synthesis of 2 studies suggests a negative association between Maternal PCB or maternal fish consumption measured during pregnancy or at birth and birthweight.	Low
Birth length							
Meta-analysis: 4; Halldorsson (2008), [40] Miyashita (2015), [36] Sagiv (2007), [35] Wohlfahrt-Veje (2014), [42]	Very serious; All studies were rated as having high or very high risk of bias particularly because of confounding; selection bias from missing data; exposure measurement bias and selective reporting of data and findings	Serious: not all the effect estimates had the same direction of effect.	Not serious	Serious; wide 95% CI for pooled effect estimates including null value. Large overall sample size (∼1600)	Undetected	The evidence suggests no association between PCB exposure during pregnancy and lactation and birth length. *r*_*p*_ = –0.04 (95% CI: –0.09, 0.02)	Very low
Head circumference							
Meta-analysis: 3; Halldorsson (2008), [40] Miyashita (2015), [36] Sagiv (2007), [35] Narrative Synthesis:1^10^; Fein (1984), [41]	Very serious; All studies were rated as having high or very high risk of bias particularly because of confounding; selection bias from missing data; exposure measurement bias and selective reporting of data and findings	Not serious	Not serious	Serious; Wide 95% CI for pooled effect estimates including null value. Large overall sample size (∼1642)	Undetected	The evidence suggests no association between PCB measured during late pregnancy and at birth and head circumference. *r*_*p*_ = –0.03 (95% CI: –0.09, 0.03) The evidence from narrative synthesis of 1 article suggests a negative association between perinatal PCB measured during late pregnancy and at birth and head circumference.	Very low
Small for gestational age							
2^10^; Mendez (2010) [39], Miyashita (2015), [36]	Extremely serious; only 2 studies with 1 being at very high risk of bias and the other with High. Risk of bias in Miyashita (2015) [23] because of confounding; selection bias from missing data; exposure measurement bias and selective reporting of data and findings	Serious; different (not significant) direction of odds ratios per each quartile of PCB exposure for SGA by weight and especially by length. More consistency in Mendez (2010) [26] although we saw occasional differences in OR directions.	Serious: 1 study did not have direct measurements of PCB.	Serious: 95% CI for the OR in 1 study included the null value. In the other study fish consumption and SGA was significant in one type of fish. The total analytic sample size was ∼932	Strongly detected; only 2 studies were included.	The evidence suggests a negative association between perinatal PCB measured during late pregnancy and at birth and small for gestational age.	Very low
Chest circumference							
1^10^; Miyashita (2015) [36],	Extremely serious; 1 study at very high risk of bias because of confounding; selection bias from missing data; exposure measurement bias and selective reporting of data and findings	Very serious; only 1 study N/A	Not serious	Very serious; wide 95% CI for beta based on *n* = 367 sample size from 1 study	Strongly detected; only 1 study with significant findings	The evidence suggests no association between perinatal PCB measured during late pregnancy and chest circumference.	Very low
Weight/change in weight							
1^10^; Wohlfahrt-Veje (2014), [42]	Very serious; only 1 study with a high risk of bias because of confounding; selection bias from missing data; and selective reporting of data and findings	Very serious; only 1 study N/A	Serious: TEQ was used as a measure of exposure in this study and can also stand for toxicity from other compounds, e.g. dioxins	Serious; Wide 95% CI for effect estimates including null value. Small sample size (*n* = 417)	Strongly detected; Only 1 study with significant findings	The evidence suggests no association between perinatal PCB measured in human milk after delivery and weight measured at 3, 18, and 36 mo	Very low
Length/change in length							
1^10^; Wohlfahrt-Veje (2014), [42]	Very serious; only 1 study with a high risk of bias because of confounding; selection bias from missing data; and selective reporting of data and findings	Very serious; only 1 study N/A	Serious; TEQ was used as a measure of exposure in this study and can also stand for toxicity from other compounds, e.g. dioxins	Not serious	Strongly detected; only 1 study with significant results for fish	The evidence suggests a positive association between Maternal PCB measured in human milk after delivery and length/height measured at 3, 18, and 36 mo	Very low

Abbreviations: *r*_*p*_, pooled partial correlation coefficient; TEQ, toxic equivalent; N/A, not applicable.

1 Grading of Recommendations, Assessment, Development, and Evaluation.

2 Downgrading domain. Response options: not serious, serious, very serious, or extremely serious. All included studies were nonrandomized studies of exposure.

3 Downgrading domain. Response options: not serious, serious, or very serious.

4 Studies were rated as “serious” if there were <3 articles and “very serious” if there were <2 articles in a particular outcome domain.

5 All studies started as serious because all domains included null results which could be an indicator of imprecision. Total sample size is the sum of sample sizes across the contributing studies. The highest sample size was considered for counting the total sample size if there were multiple articles per study.

6 Downgrading domain. Response options: undetected or strongly detected.

7 If <3 articles were included, then publication bias was automatically strongly detected because of the lack of sufficient information to confidently rule out publication bias.

8 Magnitude of effect, plausible confounding, and dose-response domains are not shown in the table because these domains were either not assessed or were “No” for all outcomes, and thus, did not provide opportunity to upgrade the evidence.

9 GRADE rating options: high, moderate, low, very low.

10 Not included in pooled partial correlation analysis. Narrative synthesis was conducted.

### Birth length

Four studies [35,36,40,42] assessed the association between PCB exposure during pregnancy and BL. One study also assessed length/height longitudinally from birth to 36 mo [42]. The *r*_*p*_ analysis revealed no correlation (*r*_*p*_ = −0.04; 95% CI: −0.09, 0.02) (Figure 2B). The findings were not different when we removed the study [35] in which the association between PCB exposure and seafood consumption was not directly assessed. We did not find evidence for significant heterogeneity (*Q*_df3_ = 3.48, *P* = 0.32; *I*^2^ = 3%). No evidence of publication bias was detected (Supplemental Figure 4). Overall, this evidence suggests no meaningful association (*r*_*p*_ = −0.04) between maternal PCB in late pregnancy and BL. ROB was rated as very serious for all studies because of confounding, selection bias because of missing data, exposure assessment, and selective reporting of results (Table 2). The overall certainty of the evidence was rated very low because of serious issues with ROB, inconsistency regarding the direction of the effect estimates, and imprecision of the *r*_*p*_ estimate with 95% CI including the null value (Table 3). The findings were similar in the sensitivity analyses when exposure to other groups of PCBs was assessed during pregnancy as described in Supplemental Method 2 (data not shown).

### Head circumference at birth

Four studies assessed the association between PCB exposure during pregnancy and HC at birth [35,36,40,41]. Three studies were included in the pooled analysis [35,36,40]. The *r*_*p*_s analysis indicated that there is no association between PCB exposure during pregnancy and HC at birth (*r*_*p*_ = −0.03; 95% CI: −0.09, 0.03) (Figure 2C). Statistical heterogeneity was not significant across the studies (*Q*_df2_ = 1.38, *P* = 0.50; *I*^2^ = 0.05%). We did not detect any evidence of publication bias (Supplemental Figure 5). The study not in the pooled analysis reported similar results [41]. The infants exposed to higher PCB concentrations had a mean 0.65 cm smaller HC compared with those with lower PCB exposure (<0.001) (Table 1). Sensitivity analyses corroborated the findings from the primary analysis (data not shown). The findings were not different when we removed the study [35] in which the association between PCB exposure and seafood consumption was not directly assessed.

Overall, evidence suggests no association between maternal PCB measured during late pregnancy and at birth and HC at birth. The certainty of evidence regarding HC was rated as very low (Table 3). Almost all studies had serious or very serious issues regarding imprecision of the pooled effect estimate, ROB because of confounding, missing data, exposure misclassification, and selective reporting of findings (TABLE 2, TABLE 3).

### Chest circumference

One article investigated the association between PCB exposure during pregnancy and CC [36]. Exposure to DL-PCBs, non-DL-PCBs, estrogenic, and antiestrogenic PCBs were each not associated with larger CC (Table 1). The evidence was graded as very low. Certainty of evidence was downgraded for ROB mainly because of confounding, selection bias because of missing data, differential measurement errors, and selective reporting of results (TABLE 2, TABLE 3). The 95% CIs included the null value, demonstrating issues with the precision of the effect estimates.

### Small for gestational age

Two studies suggested higher odds of SGA based on length and weight among infants of mothers with higher concentrations of PCB. One study [36] found that the highest quartile of PCB concentrations was associated with higher odds of SGA by weight. Although in this study, the odds of SGA based on length were lower when comparing the third and fourth (highest) quartile of maternal PCB concentrations to the first quartile, none of these associations were statistically significant (Table 1) [36]. Another study found that maternal crustacean fish intake (which was significantly associated with maternal blood PCB concentrations) was associated with significantly higher odds of SGA. Higher intake of other types of fish during pregnancy was associated with nonsignificant higher odds of SGA [39]. The certainty of evidence was rated very low for a number of reasons. The ROB in evidence stemming from 2 studies was rated to be extremely serious because of confounding, missing data, and measurement of exposure (Table 3). The indirectness was rated as serious because of the lack of direct measurement of PCB in 1 of the studies contributing to the evidence [39]. The imprecision of effect estimates was downgraded because of 95% CIs that included null values (Table 3).

### Postnatal weight and weight gain

One study [42] reported nonsignificant associations between higher PCB concentrations in maternal milk and lower weight at 3 mo but higher weight at 18 and 36 mo and greater weight gain from 0 to 18 mo [42]. The estimates for weight gain were not only positive but also not significant for 0–3 and 0–36 mo (Table 1). The ROB in this study was high because of inadequate adjustment for key confounding and selection bias because of missing data and selective reporting of findings (Table 2). The certainty of evidence is very low because of very serious ROB, and consistency of effect estimates (Table 3). We detected serious issues regarding imprecision and indirectness (Table 3).

### Postnatal length and linear growth

In a longitudinal study following children from birth to 36 mo, Wohlfahrt-Veje et al. [42] reported that although the BL was not associated with PCB, there was a significant association between PCB exposure from human milk and higher length/height change from birth to 3, 18, and 36 mo. The higher concentrations of PCB exposure during lactation were also associated with higher length/height measured at 3, 18, and 36 mo, though the significance varied (Table 1). The evidence from this study alone suggested an association between higher PCB exposure during lactation and greater long-term length/height gain. The certainty of the evidence was rated as very low because of serious and very serious issues regarding indirectness, ROB, consistency, and publication bias as the evidence was derived from only 1 study (TABLE 2, TABLE 3).

## Discussion

This review investigated the effect of PCB exposure during PL from seafood on child growth outcomes. The evidence suggests a significant but small negative association between exposure to PCB from fish intake during PL and BW (*r*_*p*_ = −0.07; 95% CI: −0.12, −0.02). No significant or meaningful association was observed with BL, CC, HC, and SGA at birth. Additionally, a positive nonsignificant association with CC at birth and significant associations with weight and length measured in 3, 18, and 36 mo were observed.

Although evidence suggests a minimal negative association between PCB exposure during pregnancy and BW, 1 included study [42] suggests that this inverse association may be attenuated and reversed during child growth, indicating that catchup growth may mitigate these adverse effects. However, accelerated growth in early childhood has been associated with the early onset of puberty [43], a higher risk of obesity [[44], [45], [46]], and cardiovascular diseases [[43], [44], [45], [46], [47]] at later life stages, outcomes left unassessed in the included study [42]. More studies are needed to confirm the association between maternal PCB exposure from seafood and BW and catchup growth.

This systematic review was designed to specifically assess contaminant exposure from seafood to inform dietary guidance. As such, our review exclusively included articles that measured both seafood and PCB exposure during PL, to analyze the relationships with each other and/or their associations with child growth outcomes (Supplemental Figure 2). Despite including fewer studies because of this focus, our findings are consistent with 2 previous systematic reviews [48,49]. These reviews reported no evidence [48] or a minor association between PCB exposure during pregnancy and BW [49]. Importantly, the distribution of PCB exposure reported in our included studies was similar to those of the other larger reviews, suggesting that our findings, though stemming from limited included studies, may be generalizable to the general population who are not exposed to very high PCB concentrations from environmental contamination.

PCBs were measured in various biological specimens, including maternal serum and blood, cord blood, and human milk collected at different time points ranging from the first trimester of pregnancy to 3 mo postpartum. Despite this variation in sampling time, these measurements likely capture PCB exposure during pregnancy and the postpartum period, given the long half-lives of PCBs, which range from 7 mo to 4 y [50,51].

We noted a small magnitude of association between PCB exposure and child growth outcomes. We attribute this small effect to a lower distribution of PCB concentrations in the populations studied (Table 1), which are predominantly from North America and Europe. In comparison, other studies have reported much higher PCB concentrations in pregnancy because of incidents such as food poisoning in Japan and Taiwan, as well as in studies reporting PCB in mixtures with other compounds such as dioxins and polychlorinated dibenzofurans [52,53]. Thus, our systematic review is generalizable to lower exposed populations, such as in North America, Japan, and Europe, and is relevant to policy decisions in similar populations [18]. On the basis of our included studies, we cannot rule out that higher levels of exposure to PCB may be associated with child growth.

Although PCB concentrations in the general human population tend to be low, the potential for these compounds to interact with others to induce negative health outcomes, including child growth, cannot be dismissed [49]. In real-world scenarios, PCBs are not found in isolation in the environment but rather as part of contaminant mixtures. The field of environmental health is transitioning from single-pollutant approaches to more holistic paradigms, exemplified by the exposome [54,55]. For example, a study of organochlorine mixtures, including PCBs, reported a stronger association between organochlorines and BW when they were part of a mixture rather than when analyzed alone [56]. Thus, co-exposure to PCB compounds and other toxicants could produce synergistic or antagonistic effects that may not be detected in our review.

The certainty of the evidence for all outcomes was low or very low because of a few limitations. Six of the included studies were at high or very high ROB. The ROB was predominantly attributable to inadequate adjustment for all key confounding factors such as socioeconomic factors (parental education or income), race, parental pre-pregnancy anthropometric measurements, nonseafood related exposure to PUFAs, or other contaminants such as dioxin-like compounds and prenatal alcohol consumption. Co-exposure to substances such as PUFAs could mask the effect of PCBs on birth outcomes [49,57]. Maternal blood PUFA concentrations were controlled in statistical models in only 2 articles [36,38]. Thus, co-exposure to substances other than PCBs could also confound the associations between exposure to PCBs and growth outcomes.

Other factors contributing to the overall high ROB included inadequate measures to address selection bias from missing observations such as multiple imputation methods, lack of standardized or validated questionnaires to assess seafood intake, and selective reporting of findings. The certainty of the evidence was also downgraded because of the lack of precision in pooled effect estimates for BL and HC at birth and lack of precision in overall effect estimates for SGA, CC at birth, and weight and length at 3, 18, and 36 mo.

Our systematic review had several strengths. Our search criteria ensured the inclusion of highly relevant studies for informing dietary guidance in HDI regions that investigated the association between PCB exposure during PL from seafood sources and growth outcomes. The concentrations of PCB exposures varied across studies, increasing the likelihood of finding an association if one truly exists and increasing external validity. Finally, we identified sufficient evidence to quantify the association between PCB exposure during pregnancy and BW, BL, and HC in a pooled analysis.

Our systematic review also has several limitations. We included articles based on whether associations between seafood and PCB concentrations were reported, rather than on the existence of such associations. Nonetheless, the majority of included studies exhibited moderate to strong correlations between maternal seafood intake and maternal PCB concentrations. Further, relevant data may not be included in this review if a study collected and reported associations between seafood intake, PCB exposure, and child outcomes but reported these data in separate publications. Additionally, we excluded gray and non-English literature which could have contributed to the limited evidence base.

## Conclusions

This review was designed to address the needs of policymakers when considering the benefits and potential harms of seafood consumption to develop appropriate dietary guidance. The evidence suggested limited or no effect of exposure to PCB from seafood intake during PL and child growth outcomes, at least at the PCB exposure distribution reported in the included studies. However, the certainty of evidence was low or very low depending on the outcome. Future research focusing on mixtures of PCBs with other contaminants to assess interactions, with multiple measurements of PCB concentrations during pregnancy and lactation as well as multiple measurements of growth outcomes with longer follow-ups could enhance our understanding of the associations between PCB exposure during PL and child growth outcomes.

## Author contributions

The authors’ contributions were as follows – MKS, AJM: designed the research; MKS, AB, RCT, RTV, SS: conducted the research; AB, RCT, RTV, SS: prepared the data; AB: synthesized and analyzed the data; AB: wrote the paper with editorial assistance from RCT, RTV, SS, MKS, AJM; and all authors approved the final manuscript. The National Academies of Sciences, Engineering, and Medicine Committee on the Role of Seafood in Child Growth and Development contributed to the analytic framework and systematic review protocol design.

## Data availability

Data described in the manuscript, code book, and analytic code will be made available upon request (for example, application and approval, payment, other).

## Conflict of interest

Texas A&M Agriculture, Food and Nutrition Evidence Center reports that financial support was provided by the National Academies of Sciences, Engineering, and Medicine. All authors declare that they have no known competing financial interests or personal relationships that could have appeared to influence the work reported in this paper.
