# Supplementary material for: Associations Between Maternal Polychlorinated Biphenyls (PCBs) Exposure from Seafood Consumption during Pregnancy and Lactation and Child Growth: A Systematic Review and Meta-Analysis
Source: Adv Nutr. 2024 Nov 30;16(1):100350. doi: 10.1016/j.advnut.2024.100350 (PMC11784771; doi:10.1016/j.advnut.2024.100350)
Supplement: multimedia component 1 [file mmc1.docx]

**Supplemental Online Content**

**Associations Between Maternal Polychlorinated Biphenyls (PCBs) Exposure from Seafood Consumption and Child Growth: A Meta-Analysis**

Contents

[Supplementary Method 1. Methods to Calculate Partial Correlations 3](#_Toc183260793)

[Supplementary Method 2. Methods to Conduct Sensitivity Analysis 4](#_Toc183260794)

[Supplementary Figure 1: Analytical PICO Framework for a Systematic Review of the Association Between Polychlorinated Biphenyls from Seafood Consumption During Pregnancy and Lactation and Child Growth 5](#_Toc183260795)

[Supplementary Figure 2. Inclusion Criteria based on seafood intake and PCB exposure 6](#_Toc183260796)

[Supplementary Figure 3: Funnel Plots of Studies Included in the Pooled Analysis of Perinatal Exposure to PCBs and Birth Weight 7](#_Toc183260797)

[Supplementary Figure 4: Funnel Plots of Studies Included in the Pooled Analysis of Perinatal Exposure to PCBs and Birth Length 8](#_Toc183260798)

[Supplementary Figure 5: Funnel Plots of Studies Included in the Pooled Analysis of Perinatal Exposure to PCBs and Head Circumference 9](#_Toc183260799)

[Supplementary Table 1a: PRISMA 2020 Main Checklist 10](#_Toc183260800)

[Supplementary Table 1b: PRIMSA Abstract Checklist 17](#_Toc183260801)

[Supplementary Table 2: Search Strategy for a Systematic Review of the Association Between Polychlorinated Biphenyls From Seafood Consumption During Pregnancy and Lactation and Child Growth 19](#_Toc183260802)

[Supplementary Table 3: Inclusion and Exclusion Criteria for a Systematic Review of the Association Between Polychlorinated Biphenyls From Seafood Consumption During Pregnancy and Lactation and Child Growth 22](#_Toc183260803)

[Supplementary Table 4: The Relationship Between Seafood and PCB Concentrations in Articles with Data Availability 24](#_Toc183260804)

# Supplementary Method 1. Methods to Calculate Partial Correlations

Partial correlations and their corresponding variances were calculated from reported estimates of effects and corresponding t-values using the following equations:

$$r_{p}=\frac{t_{predictor}}{\sqrt{t_{predictor}^{2}+df}}$$

$$var\left( r_{p} \right)=\frac{{(1-r_{p}^{2})}^{2}}{n-p-1}$$

Where r_p_ is the partial correlation coefficient, t_predictor_ is the t-value of predictor of interest derived from multiple regression analysis and df is the degrees of freedom of the regression model of interest. The r_p_ for each specific study would be interpreted as the correlation estimate between PCB concentrations and specific birth outcomes after the correlations between the rest of the predictors and the outcome is partialled out.^11^ Whenever the t-value was not available, it was calculated by dividing the effect estimate(b) by its corresponding standard error (SE). The variance of r_p_ was calculated using equation-II, where n is the analytic sample size, and p is the total number of predictors used in the regression model of interest.

# Supplementary Method 2. Methods to Conduct Sensitivity Analysis

For a sensitivity analysis, we used the effect estimate values with lowest magnitude to calculate partial correlations to explore if the pooled partial correlation estimate was sensitive to the choice of effect estimates in individual studies. We also excluded one study from birth weight (BW) and birth length (BL) pooled analysis where the partial correlation coefficients were calculated based on standardized effect estimates. We replaced the effect estimates for DL-PCBs with effect estimates for non-dioxin-like PCBs (NDL-PCBs) and estrogenic PCBs (E-PCBs) to examine if our findings were sensitive to selection of PCB groups.

# Supplementary Figure 1: Analytical PICO Framework for a Systematic Review of the Association Between Polychlorinated Biphenyls from Seafood Consumption During Pregnancy and Lactation and Child Growth


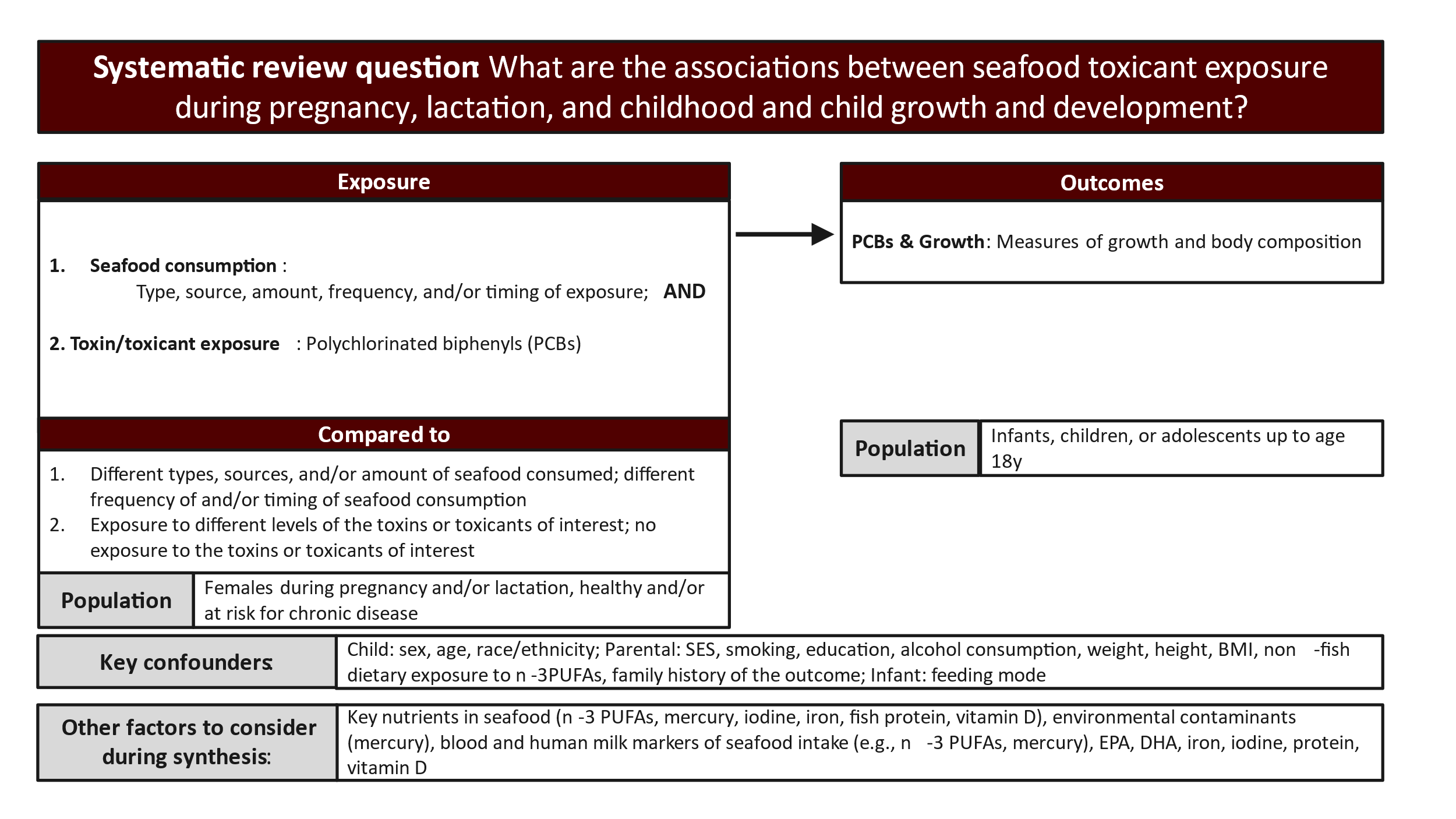


# Supplementary Figure 2. Inclusion Criteria based on seafood intake and PCB exposure

 
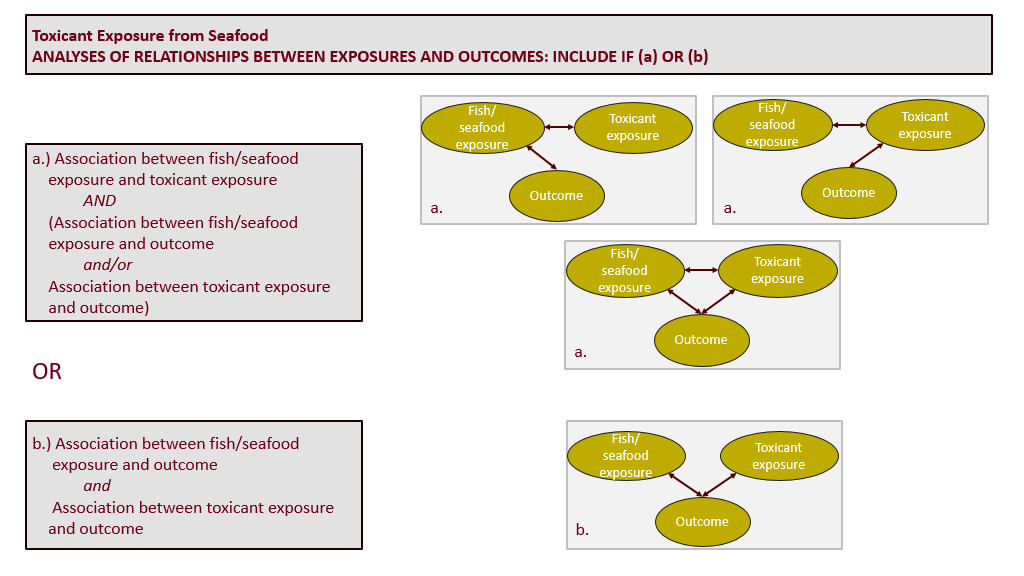


# Supplementary Figure 3: Funnel Plots of Studies Included in the Pooled Analysis of Perinatal Exposure to PCBs and Birth Weight


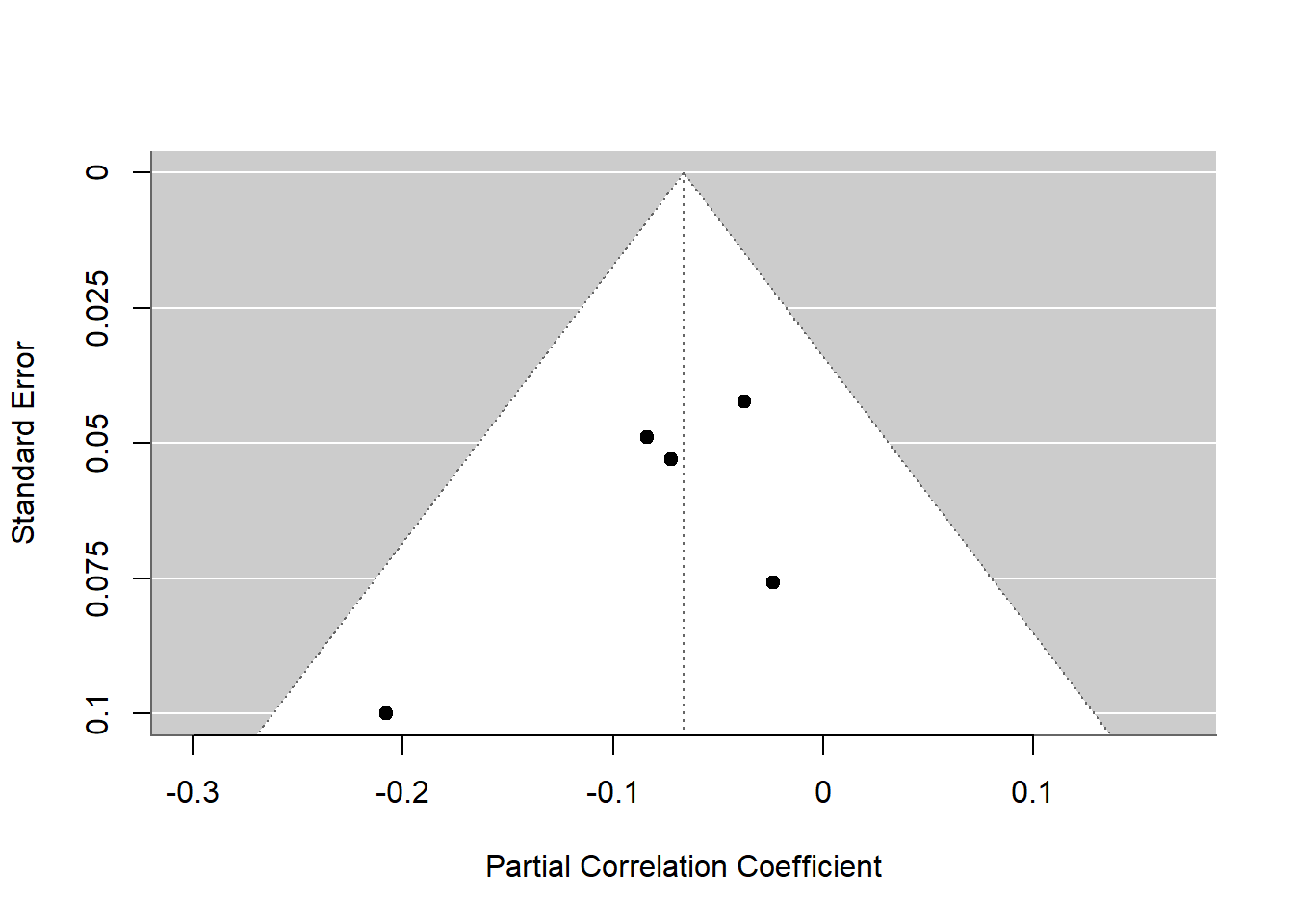


# Supplementary Figure 4: Funnel Plots of Studies Included in the Pooled Analysis of Perinatal Exposure to PCBs and Birth Length


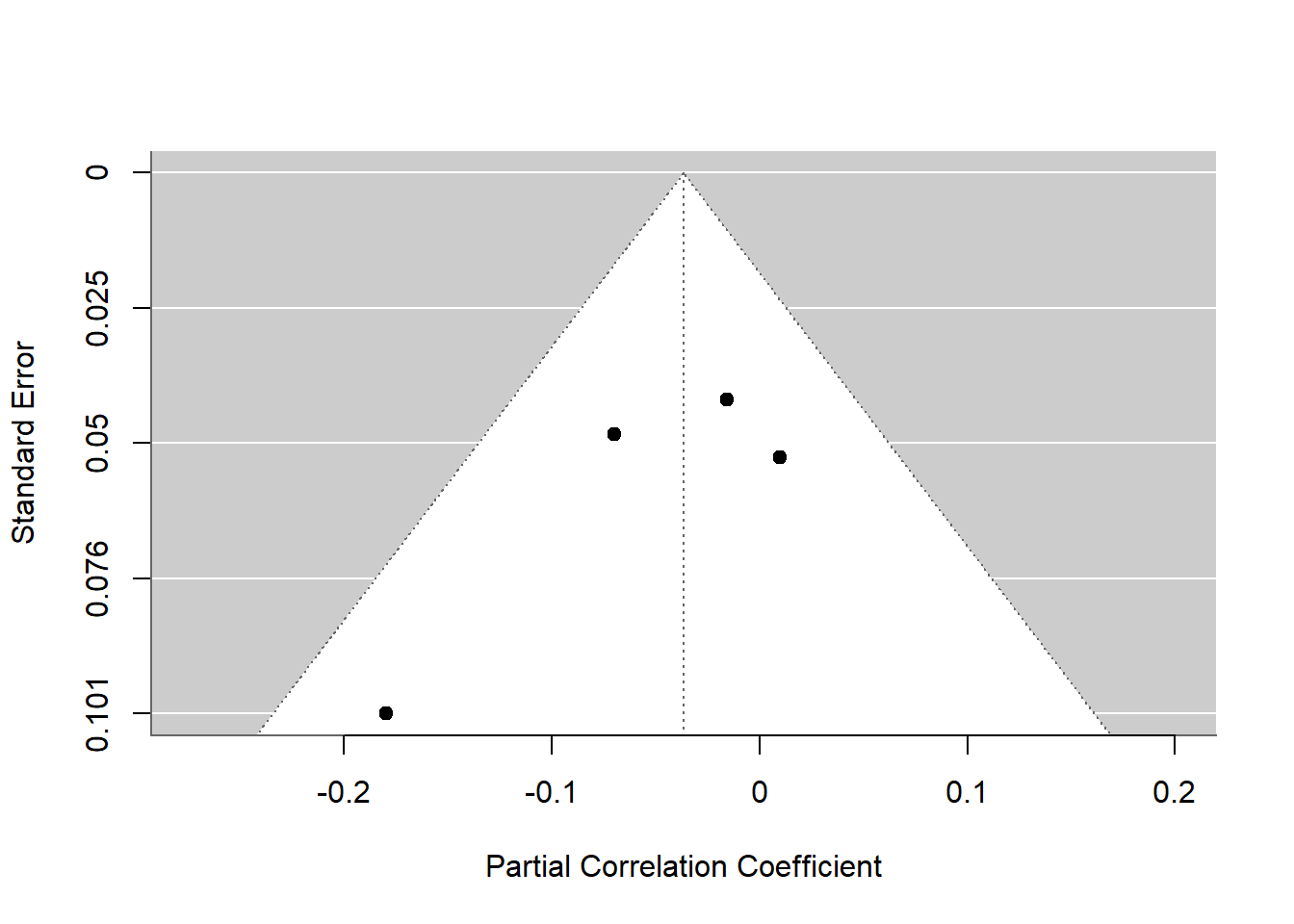


# Supplementary Figure 5: Funnel Plots of Studies Included in the Pooled Analysis of Perinatal Exposure to PCBs and Head Circumference


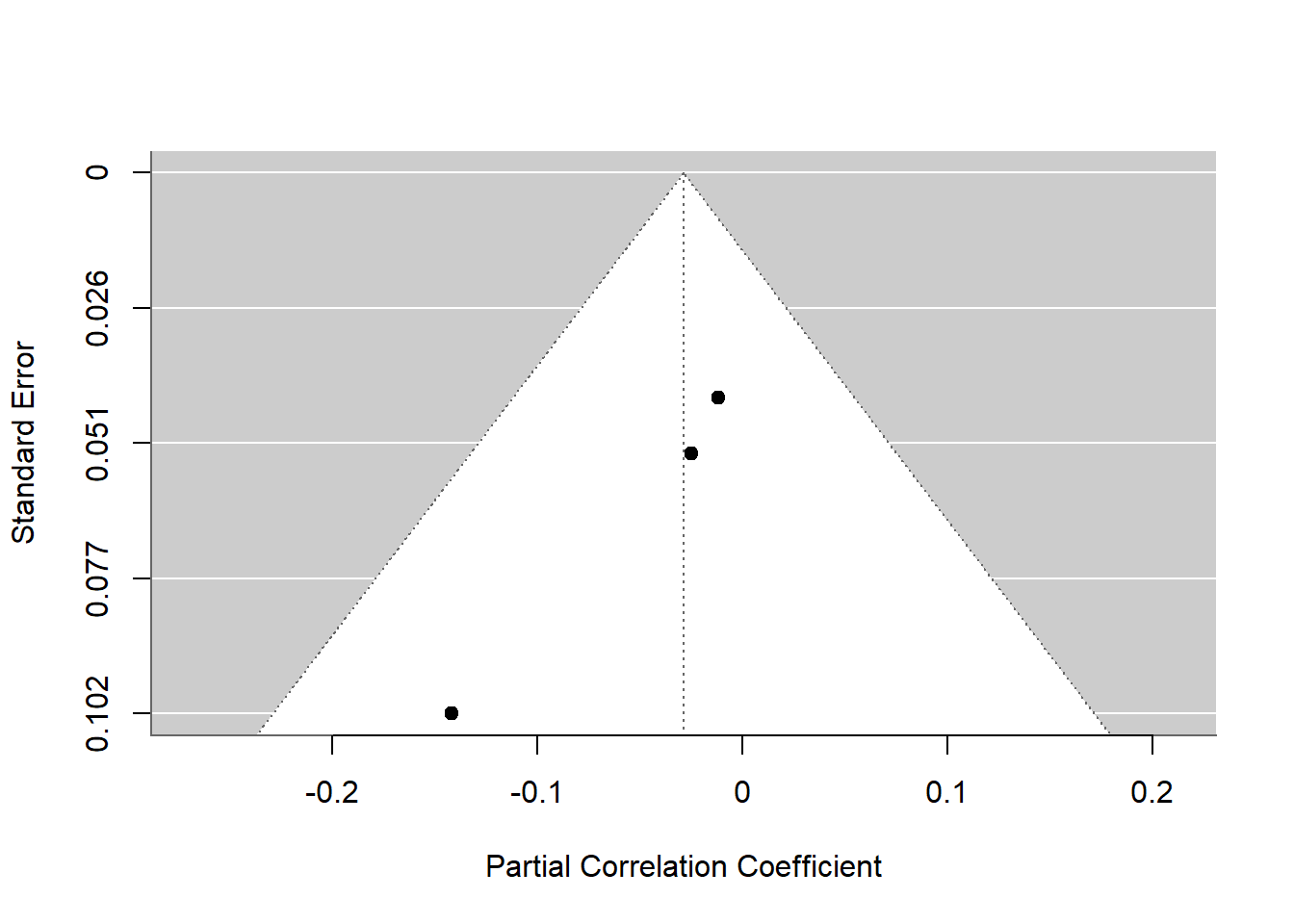


# Supplementary Table 1a: PRISMA 2020 Main Checklist

| **Topic** | **No.** | **Item** | **Location where item is reported** |
| --- | --- | --- | --- |
| **TITLE** |  |  |  |
| **Title** | 1 | Identify the report as a systematic review. | 1 |
| **ABSTRACT** |  |  |  |
| **Abstract** | 2 | See the PRISMA 2020 for Abstracts checklist. | 3 |
| **INTRODUCTION** |  |  |  |
| **Rationale** | 3 | Describe the rationale for the review in the context of existing knowledge. | 4 |
| **Objectives** | 4 | Provide an explicit statement of the objective(s) or question(s) the review addresses. | 4 |
| **METHODS** |  |  |  |
| **Eligibility criteria** | 5 | Specify the inclusion and exclusion criteria for the review and how studies were grouped for the syntheses. | 4,5 Supplementary Table2,3 |
| **Information sources** | 6 | Specify all databases, registers, websites, organizations, reference lists and other sources searched or consulted to identify studies. Specify the date when each source was last searched or consulted. | 4 |
| **Search strategy** | 7 | Present the full search strategies for all databases, registers and websites, including any filters and limits used. | Supplementary Table2 |
| **Selection process** | 8 | Specify the methods used to decide whether a study met the inclusion criteria of the review, including how many reviewers screened each record and each report retrieved, whether they worked independently, and if applicable, details of automation tools used in the process. | page 5, Supplementary Table3 |
| **Data collection process** | 9 | Specify the methods used to collect data from reports, including how many reviewers collected data from each report, whether they worked independently, any processes for obtaining or confirming data from study investigators, and if applicable, details of automation tools used in the process. | page 5 |
| **Data items** | 10a | List and define all outcomes for which data were sought. Specify whether all results that were compatible with each outcome domain in each study were sought (e.g., for all measures, time points, analyses), and if not, the methods used to decide which results to collect. | page 5 |
|  | 10b | List and define all other variables for which data were sought (e.g., participant and intervention characteristics, funding sources). Describe any assumptions made about any missing or unclear information. | page 5 |
| **Study risk of bias assessment** | 11 | Specify the methods used to assess risk of bias in the included studies, including details of the tool(s) used, how many reviewers assessed each study and whether they worked independently, and if applicable, details of automation tools used in the process. | page 5 |
| **Effect measures** | 12 | Specify for each outcome the effect measure(s) (e.g., risk ratio, mean difference) used in the synthesis or presentation of results. | page 5, eMethods 1 |
| **Synthesis methods** | 13a | Describe the processes used to decide which studies were eligible for each synthesis (e.g., tabulating the study intervention characteristics and comparing against the planned groups for each synthesis (item 5)). | page 5, eMethods 1 |
|  | 13b | Describe any methods required to prepare the data for presentation or synthesis, such as handling of missing summary statistics, or data conversions. | page 5, eMethods 1 |
|  | 13c | Describe any methods used to tabulate or visually display results of individual studies and syntheses. | page 5, eMethods 1 |
|  | 13d | Describe any methods used to synthesize results and provide a rationale for the choice(s). If meta-analysis was performed, describe the model(s), method(s) to identify the presence and extent of statistical heterogeneity, and software package(s) used. | page 5, eMethods 1 |
|  | 13e | Describe any methods used to explore possible causes of heterogeneity among study results (e.g., subgroup analysis, meta-regression). | page 5 |
|  | 13f | Describe any sensitivity analyses conducted to assess robustness of the synthesized results. |  |
| **Reporting bias assessment** | 14 | Describe any methods used to assess risk of bias due to missing results in a synthesis (arising from reporting biases). | page 5 |
| **Certainty assessment** | 15 | Describe any methods used to assess certainty (or confidence) in the body of evidence for an outcome. | page 5 |
| **RESULTS** |  |  |  |
| **Study selection** | 16a | Describe the results of the search and selection process, from the number of records identified in the search to the number of studies included in the review, ideally using a flow diagram. | Figure 1, page 6 |
|  | 16b | Cite studies that might appear to meet the inclusion criteria, but which were excluded, and explain why they were excluded. | N/A |
| **Study characteristics** | 17 | Cite each included study and present its characteristics. | Table 1 |
| **Risk of bias in studies** | 18 | Present assessments of risk of bias for each included study. | Table 2 |
| **Results of individual studies** | 19 | For all outcomes, present, for each study: (a) summary statistics for each group (where appropriate) and (b) an effect estimates and its precision (e.g., confidence/credible interval), ideally using structured tables or plots. | Table 1, Figure 2 |
| **Results of syntheses** | 20a | For each synthesis, briefly summarize the characteristics and risk of bias among contributing studies. | Table 1, Table 2 |
|  | 20b | Present results of all statistical syntheses conducted. If meta-analysis was done, present for each the summary estimate and its precision (e.g., confidence/credible interval) and measures of statistical heterogeneity. If comparing groups, describe the direction of the effect. | Figure 2 |
|  | 20c | Present results of all investigations of possible causes of heterogeneity among study results. | pages 6-8 |
|  | 20d | Present results of all sensitivity analyses conducted to assess the robustness of the synthesized results. | pages 6-8 |
| **Reporting biases** | 21 | Present assessments of risk of bias due to missing results (arising from reporting biases) for each synthesis assessed. | Table 4 |
| **Certainty of evidence** | 22 | Present assessments of certainty (or confidence) in the body of evidence for each outcome assessed. | Table 3 |
| **DISCUSSION** |  |  |  |
| **Discussion** | 23a | Provide a general interpretation of the results in the context of other evidence. | pages 8.9 |
|  | 23b | Discuss any limitations of the evidence included in the review. | page 9 |
|  | 23c | Discuss any limitations of the review processes used. | page 9 |
|  | 23d | Discuss implications of the results for practice, policy, and future research. | page 10 |
| **OTHER INFORMATION** |  |  |  |
| **Registration and protocol** | 24a | Provide registration information for the review, including register name and registration number, or state that the review was not registered. | page 4 |
|  | 24b | Indicate where the review protocol can be accessed, or state that a protocol was not prepared. | page 4 |
|  | 24c | Describe and explain any amendments to information provided at registration or in the protocol. | N/A |
| **Support** | 25 | Describe sources of financial or non-financial support for the review, and the role of the funders or sponsors in the review. | page 11 |
| **Competing interests** | 26 | Declare any competing interests of review authors. |  |
| **Availability of data, code, and other materials** | 27 | Report which of the following are publicly available and where they can be found template data collection forms; data extracted from included studies; data used for all analyses; analytic code; any other materials used in the review. | N/A |

# Supplementary Table 1b: PRIMSA Abstract Checklist

| **Topic** | **No.** | **Item** | **Reported?** |
| --- | --- | --- | --- |
| **TITLE** |  |  |  |
| **Title** | 1 | Identify the report as a systematic review. | Yes |
| **BACKGROUND** |  |  |  |
| **Objectives** | 2 | Provide an explicit statement of the main objective(s) or question(s) the review addresses. | Yes |
| **METHODS** |  |  |  |
| **Eligibility criteria** | 3 | Specify the inclusion and exclusion criteria for the review. | Yes |
| **Information sources** | 4 | Specify the information sources (e.g., databases, registers) used to identify studies and the date when each was last searched. | Yes |
| **Risk of bias** | 5 | Specify the methods used to assess risk of bias in the included studies. | Yes |
| **Synthesis of results** | 6 | Specify the methods used to present and synthesize results. | Yes |
| **RESULTS** |  |  |  |
| **Included studies** | 7 | Give the total number of included studies and participants and summarize relevant characteristics of studies. | Yes |
| **Synthesis of results** | 8 | Present results for main outcomes, preferably indicating the number of included studies and participants for each. If meta-analysis was done, report the summary estimate and confidence/credible interval. If comparing groups, indicate the direction of the effect (i.e., which group is favored). | Yes |
| **DISCUSSION** |  |  |  |
| **Limitations of evidence** | 9 | Provide a brief summary of the limitations of the evidence included in the review (e.g., study risk of bias, inconsistency, and imprecision). | Yes |
| **Interpretation** | 10 | Provide a general interpretation of the results and important implications. | Yes |
| **OTHER** |  |  |  |
| **Funding** | 11 | Specify the primary source of funding for the review. | No |
| **Registration** | 12 | Provide the register name and registration number. | No |

From: Page MJ, McKenzie JE, Bossuyt PM, Boutron I, Hoffmann TC, Mulrow CD, et al. The PRISMA 2020 statement: an updated guideline for reporting systematic reviews. MetaArXiv. 2020, September 14. DOI: 10.31222/osf.io/v7gm2. For more information, visit: [www.prisma-statement.org](http://www.prisma-statement.org)

# Supplementary Table 2: Search Strategy for a Systematic Review of the Association Between Polychlorinated Biphenyls From Seafood Consumption During Pregnancy and Lactation and Child Growth

Database: Medline

Provider: Ovid

Date(s) searched: 12/21/2023

Date range searched: no year limits

Limits: no language or year limits

Search terms:

| 1 | exp Fishes/ or exp Fish Proteins/ or exp seafood/ or exp fish products/ or exp fish proteins, dietary/ or exp shellfish/ | 241107 |
| --- | --- | --- |
| 2 | (seafood or sea foods or sea food or sea-food or sea-foods or fish consumption or fishes or fish protein or fish proteins or fish product or fish products or fish meal or fish flour or fatty fish or shellfish or shellfish proteins or mercurialism or mercury poisoning or methylmercury or sharks or swordfish or tuna or salmonine or salmon or sardine* or sardines or gadiformes or pollock or flounder or cod or tilapia or shrimp or oyster or oysters or clams or scallops or crab or crabs or perciformes or mackerel or catfishes or trout or lobster or decapodiformes or squid or halibut or mahi mahi or crayfish or crawfish or anchovy or herring or rockfish or marine product or marine products or marlin or orange roughy or tile fish or whales or perch or walleye fish or lake trout or salmonid or catfish or sushi or cerviche or sashimi or gravlax or tuna tartare or seafood crudo or fluke crudo or fluke or carpaccio or e-la-ota or poke or hinava or gohu ikan or esqueixada or kelaguen or namero or kilawin or stroganina or yusheng or koi pla or kokoda or kuai or lakerda or larb pla or ota ika or tiradito or xato or umai or salmo salar or atlantic salmon or salmonids).ti,ab,kw. | 200392 |
| 3 | or/1-2 | 359787 |
| 4 | exp Pregnancy/ or exp Pregnancy Complications/ or exp Pregnancy Outcome/ | 1053515 |
| 5 | (prenatal* or postnatal* or antenatal* or maternal or pregnan*).ti,ab. | 923134 |
| 6 | exp lactation/ | 48670 |
| 7 | (lactat* or breastfeed* or nursing).ti,ab. | 517219 |
| 8 | or/4-7 | 1844334 |
| 9 | exp polychlorinated biphenyl/ | 17656 |
| 10 | (polychlorobiphenyl* or polychlorobiphenyl* or pcb or phenoclor or "kc 400" or "clophen c" or (polychlorinated adj1 (biphenyl* or diphenyl*))).ti,ab. | 21077 |
| 11 | or/9-10 | 24810 |
| 12 | 3 and 8 and 11 | 368 |
| 13 | (animals not humans).sh. | 5146063 |
| 14 | (mice or mouse or rat or rats).ti,ab. | 2833100 |

Database: Embase

Provider: Ovid

Date(s) searched: 12/21/2023

Date range searched: no year limits

Limits: no language or year limits

 Search terms:

| 1 | exp sea food/ or exp fish/ or exp fish consumption/ or exp fish protein/ or exp fish product/ or exp fish flour/ or exp shellfish/ or exp shellfish protein/ or exp mercurialism/ or exp methylmercury/ or exp shark/ or exp swordfish/ or exp tuna/ or exp salmonine/ or exp sardine/ or exp gadiformes/ or exp flounder/ or exp atlantic cod/ or exp cod/ or exp tilapia/ or exp shrimp/ or exp oyster/ or exp mya/ or exp bivalve/ or exp clam/ or exp scallop/ or exp brachyura/ or exp crab/ or exp perciformes/ or exp mackerel/ or exp lobster/ or exp decapodiformes/ or exp squid/ or exp halibut/ or exp crayfish/ or exp anchovy/ or exp herring/ or exp rockfish/ or exp cetacea/ or exp perch/ or exp bass/ or exp lake trout/ or exp catfish/ or exp sushi/ or exp salmo salar/ or exp salmonid/ | 325671 |
| --- | --- | --- |
| 2 | (seafood or sea foods or sea food or sea-food or sea-foods or fish consumption or fishes or fish protein or fish proteins or fish product or fish products or fish meal or fish flour or fatty fish or shellfish or shellfish proteins or mercurialism or mercury poisoning or methylmercury or sharks or swordfish or tuna or salmonine or salmon or sardine* or sardines or gadiformes or pollock or flounder or cod or tilapia or shrimp or oyster or oysters or clams or scallops or crab or crabs or perciformes or mackerel or catfishes or trout or lobster or decapodiformes or squid or halibut or mahi mahi or crayfish or crawfish or anchovy or herring or rockfish or marine product or marine products or marlin or orange roughy or tile fish or whales or perch or walleye fish or lake trout or salmonid or catfish or sushi or cerviche or sashimi or gravlax or tuna tartare or seafood crudo or fluke crudo or fluke or carpaccio or e-la-ota or poke or hinava or gohu ikan or esqueixada or kelaguen or namero or kilawin or stroganina or yusheng or koi pla or kokoda or kuai or lakerda or larb pla or ota ika or tiradito or xato or umai or salmo salar or atlantic salmon or salmonids).ti,ab,kw. | 207264 |
| 3 | or/1-2 | 415213 |
| 4 | exp pregnant woman/ or exp pregnancy complication/ or exp pregnancy outcome/ | 631778 |
| 5 | (prenatal* or postnatal* or antenatal* or maternal or pregnan*).ti,ab. | 1123637 |
| 6 | exp lactation/ | 57079 |
| 7 | (lactat* or breastfeed* or nursing).ti,ab. | 572176 |
| 8 | or/4-7 | 1876271 |
| 9 | exp polychlorinated biphenyl/ | 22489 |
| 10 | (polychlorobiphenyl* or polychlorobiphenyl* or pcb or phenoclor or "kc 400" or "clophen c" or (polychlorinated adj1 (biphenyl* or diphenyl*))).ti,ab. | 24337 |
| 11 | or/9-10 | 30479 |
| 12 | 3 and 8 and 11 | 528 |
| 13 | (animal$ not human$).sh,hw. | 4655686 |
| 14 | (mice or mouse or rat or rats).ti,ab. | 3271184 |
| 15 | 12 not (13 or 14) | 362 |

Database: Cochrane CENTRAL

Provider: Wiley

Date(s) searched: 2/15/2024

Date range searched: no year limits

Limits: no language or year limits

 Search terms:

| #1 | (seafood or sea foods or sea food or sea-food or sea-foods or fish consumption or fishes or fish protein or fish proteins or fish product or fish products or fish meal or fish flour or fatty fish or shellfish or shellfish proteins or mercurialism or mercury poisoning or methylmercury or sharks or swordfish or tuna or salmonine or salmon or sardine* or sardines or gadiformes or pollock or flounder or cod or tilapia or shrimp or oyster or oysters or clams or scallops or crab or crabs or perciformes or mackerel or catfishes or trout or lobster or decapodiformes or squid or halibut or mahi mahi or crayfish or crawfish or anchovy or herring or rockfish or marine product or marine products or marlin or orange roughy or tile fish or whales or perch or walleye fish or lake trout or salmonid or catfish or sushi or cerviche or sashimi or gravlax or tuna tartare or seafood crudo or fluke crudo or fluke or carpaccio or e-la-ota or poke or hinava or gohu ikan or esqueixada or kelaguen or namero or kilawin or stroganina or yusheng or koi pla or kokoda or kuai or lakerda or larb pla or ota ika or tiradito or xato or umai or salmo salar or atlantic salmon or salmonids).ti,ab,kw. |
| --- | --- |
| #2 | (prenatal* or postnatal* or antenatal* or maternal or pregnan*).ti,ab. |
| #3 | (lactat* or breastfeed* or nursing).ti,ab. |
| #4 | (polychlorobiphenyl* or polychlorobiphenyl* or pcb or phenoclor or "kc 400" or "clophen c") or (polychlorinated adj1 (biphenyl* or diphenyl*)) |
|  | 0 results- limited to Cochrane Central |

# Supplementary Table 3: Inclusion and Exclusion Criteria for a Systematic Review of the Association Between Polychlorinated Biphenyls From Seafood Consumption During Pregnancy and Lactation and Child Growth

| Category | Inclusion Criteria | Exclusion Criteria |
| --- | --- | --- |
| Population:   - Country - Health status - Exp pop - Outcome pop - Species | ● Country: Individuals living in countries ranked as high or very high on the Human Development Index during the study.  ● Exposed population: Individuals in the general population who are pregnant or lactating, infants, children, or adolescents up to age 18 years.  ● Outcome population: Children and adolescents (up to age 18 years).  ● Species: Humans only | ● Health status: Studies exclusively of participants with a chronic condition, hospitalized with an illness or injury. Examples include:   - Diabetes (not including gestational diabetes) - Cancer - Cardiometabolic disorders - Chronic kidney disease - Malabsorption (any disorder that causes malabsorption from the gastrointestinal tract) - Asthma   ● Species: Non-human |
| Exposure:  Seafood intake + Toxicant | Must contain Exposure 1 AND Exposure 2  **Exposure 1: Seafood consumption:**  ● Types (e.g., salmon, tuna, bass)  ● Sources (e.g., sea, fresh water, farmed, canned, wild)  ● Amount (e.g., ounces per day, grams per meal)  ● Frequency (e.g., daily, twice a week)  ● Duration (e.g., length of time consuming seafood)  ● Preparation (e.g., fried, baked)  ● Timing (e.g., by trimester, age)    **Exposure 2: Toxin or toxicants**  ○ Polychlorinated biphenyls (PCBs), dioxin and dioxin-like compounds (DLCs) | ● Studies that do not report on toxicant exposure in fish AND seafood consumption  ● Supplements  ● Infant formula  **● Toxins from algal blooms**   - **Cyanobacteria** - **Ciguatera** - **Scombroid** - **Domoic acid (red algae)**   **● Microorganisms (hepatitis, salmonella, e coli)** |
| Comparator | ● Exposure to different levels of the toxins or toxicants of interest; No exposure to the toxins or toxicants of interest  ● Different types, sources, amounts, frequencies, durations, preparations, or timings of seafood consumption; No seafood consumption | ● No comparator |
| Outcomes:  ANY Adverse effect | Growth-Related   - Measures of growth and body composition - Failure to thrive (malnutrition, protein deficiency) |  |
| Study designs | - Randomized controlled trials - Controlled (nonrandomized) trials - Cohort (observational) studies, prospective or retrospective - Case-cohort studies (Nested case-control) - Case-control studies - Before-after studies | - **Cross-sectional studies** - Studies without primary data, such as systematic reviews, narrative reviews, editorials, and commentaries - Case reports - Studies reported in theses or conference abstracts only - Studies not reported in English |

# Supplementary Table 4: The Relationship Between Seafood and PCB Concentrations in Articles with Data Availability

| **Article ID, Cohort name, Country** | **Results** | **Findings Summary** |
| --- | --- | --- |
| Fein, 1984; Lake Michigan Cohort Study; United States | r^a^, p-value  r=0.37, p<0.001  Correlation maternal blood PCB levels and cord blood PCB levels:  r=0.41, p<0.001 | High fish consumption correlated with overall higher PCB levels. |
| Grandjean, 2001; Faroe Island cohort study; Faroe Islands | NR | Fish, whale meat, and blubber consumption best predictor of PCB concentrations |
| Halldorsson, 2008; Danish National Birth Cohort; Denmark | β (95% CI)  β =% Increase in PCB concentrations per fatty fish meal consumption per month  Fatty fish intake (meals/mo)  0: 0 (ref)  1-3: 3.0(-9.0, 15.0)  ≥4: 18.0 (5.0, 34.0)  P for trend: 0.005 | Significant increase in maternal plasma PCB concentration for ≥4 of fatty fish consumption per month |
| Mendez, 2010; INfancia y Medio Ambiente: ‘INMA’; Spain | PCB (p-value for difference by likelihood ratio tests); Geometric Mean (% detectable by fish intake servings per week) ng/ml  PCB 138 (p=0.005):  <=3 servings per week: 0.08 (60.5%)  >3-6 servings per week :0.10 (75.7%)  >6 servings per week :0.11 (81.5 %) | Higher PCB concentrations in maternal serum per higher servings of seafood per week. |
|  | PCB 153(p=0.001):  <=3 servings per week: 0.13 (91.7%)  >3-6 servings per week: 0.19 (93.1%)  >6 servings per week: 0.20 (93.6%) |  |
|  | PCB 180 (p=0.004):  <=3 servings per week: 0.10 (82.1%)  >3-6 servings per week: 0.12 (81.2%)  >6 servings per week: 0.13 (85.8%) |  |
| Miyashita, 2015; Hokaido Study on Environment and Children's health; Japan | Median (min, max)  Shoreline fish: NS, p-value NR  < Once/wk: 101 (17.8, 362)  ≥Once/wk: 113 (19.6, 495) | Association between fish consumption during pregnancy and maternal total PCBs |
|  | Median (min, max)  Pelagic fish: NS, p-value NR  < Once/wk: 106 (17.8, 362)  ≥Once/wk: 109 (27.4, 495) |  |
|  | r^a^, p-value  Fish intake (g/d): 0.187, p<0.01  Fatty fish intake (g/d): 0.141, p<0.01  Shellfish intake (g/d): 0.087, NS, p=NR | Overall significant correlation between fish consumption at delivery and maternal total PCBs |
| Sagiv, 2007; New Bedford Cohort Study; United States | NR | NR |
| Wohlfahrt-Veje, 2014; Copenhagen Mother Child Cohort of Growth and Reproduction | mean (median)  No/Seldom: 17.8 (16.6)  >1/mo: 21.6 (20.3)  P < 0.005 | Significantly higher average TEQ in breastmilk of mothers who consumed seafood more than once in month |

Abbreviations: g/d, gram per day; mo, month; NR, Not Reported; NS, Not significant; TEQ, Toxic equivalent; wk, week

^a^ Spearman rank correlation coefficient
